# Spatial patterns and prognostic relevance of CD1a^+^ immature and CD208^+^ mature dendritic cells in colorectal cancer from non-tumor adjacent mucosa to liver metastases

**DOI:** 10.1007/s00262-025-04238-2

**Published:** 2025-12-18

**Authors:** Sergii Pavlov, Esraa Ali, Wenjing Ye, Lenka Červenková, Filip Ambrozkiewicz, Ondřej Vyčítal, Ondřej Daum, Václav Liška, Kari Hemminki, Andriy Trailin

**Affiliations:** 1 Laboratory of Translational Cancer Genomics, Faculty of Medicine in Pilsen, Biomedical Center, Charles University, Alej Svobody 1665/76, 32300 Pilsen, Czech Republic; 2 Laboratory of Cancer Treatment and Tissue Regeneration, Faculty of Medicine in Pilsen, Biomedical Center, Charles University, Alej Svobody 1665/76, 32300 Pilsen, Czech Republic; 3 Department of Surgery and Biomedical Center, Faculty of Medicine in Pilsen, Charles University, Alej Svobody 80, 32300 Pilsen, Czech Republic; 4 The Department of Pathology, Regional Hospital Liberec, Husova 1430/34, 460 01 Liberec, Liberec - Stare Mesto, Czech Republic; 5 Department of Cancer Epidemiology, German Cancer Research Center, Im Neuenheimer Feld 280, 69120 Heidelberg, Germany

**Keywords:** Primary colorectal cancer, Synchronous and metachronous liver metastases, Immature and mature dendritic cells, Spatial immune profiling, Overall survival

## Abstract

The prognostic role of dendritic cells (DCs) in colorectal cancer (CRC) and paired liver metastases (LM) remains unclear, particularly regarding the dynamics of immature CD1a^+^ and mature CD208^+^ subsets across anatomical compartments and synchronous versus metachronous disease. Patients and methods. This retrospective cohort included patients undergoing resection of primary CRC (pCRC) and synchronous LM (*N* = 55) or metachronous LM (*N* = 44). Immunohistochemical staining for CD1a and CD208 was performed on non-tumor adjacent mucosa (NAM), as well as tumor center (TC), inner margin (IM), outer margin (OM), and peritumoral zone (PT) of both pCRC and LM. Cell densities were quantified on whole-slide images using QuPath software and correlated with overall survival (OS). Results. CD1a^+^ DCs were nearly absent in NAM but enriched in pCRC TC, whereas CD208^+^ DCs predominated in lymphoid aggregates associated with NAM and peripheral compartments of pCRC and LM. CD1a^+^ cells followed a TC/IM > OM/PT gradient, while CD208^+^ cells showed the opposite, consistent with a recruitment–maturation axis. In synchronous cases, CD1a^+^ densities were higher in pCRC than LM, supporting the role of the primary tumor as a “monocyte reservoir.” Survival analysis revealed that high CD208^+^ density in TC of synchronous LM (hazard ratio (HR) = 0.47; *p* = 0.033) and high CD1a^+^ density in TC of metachronous LM (HR = 0.44; *p* = 0.050) were both associated with reduced mortality risk. Conclusion. This study provides the first detailed mapping of CD1a^+^ and CD208^+^ DCs across NAM, pCRC and paired LM, indicating that their prognostic impact is determined not only by absolute numbers but, more importantly, by compartmental distribution and the temporal pattern of metastasis.

**Supplementary Information:**

The online version contains supplementary material available at 10.1007/s00262-025-04238-2.

## Introduction

Colorectal cancer (CRC) is a leading cause of cancer-related deaths. Despite improved treatment, up to 60% of patients ultimately develop distant metastases, the liver being the main site because of portal drainage and a permissive pre‑metastatic niche [1]. The immune contexture of tumor microenvironment (TME) in both primary and metastatic tumors is now recognized as a key determinant of whether disseminated CRC cells are eliminated, held in dormancy, or allowed to progress [2–4].

Among the diverse immune populations shaping the CRC microenvironment, dendritic cells (DCs) represent central orchestrators of antitumor immunity [5]. DCs are a highly heterogeneous cell population comprised of several subsets with distinct origins, locations, migratory, and functional properties [4], which bridge innate and adaptive immune responses [6].

First subset of classical DCs (cDC1) is the principal cells for cross-presenting tumor antigens to CD8^+^ T-lymphocytes and initiating cytotoxic responses, besides priming CD4^+^ T-cells [7]. The cDC2 subset activates CD4^+^ T-lymphocytes and drives their polarization into diverse T-helper populations [8]. The contribution of cDC1 and cDC2 to antitumor immunity is context-dependent and often contradictory, complicating evaluation of their prognostic impact in CRC [8]. Plasmacytoid DCs (pDCs) are characterized by robust production of type I interferons and, under physiological conditions, play a key role in antiviral defense [9]. It is known that pDCs can infiltrate solid tumors, although they are relatively scarce [10] and their antitumor efficacy is limited [10]. Monocytic DCs (moDCs) arise from circulating monocytes during precancerous transformation and are dominant in tumor tissue [11]. Although moDs can activate CD4^+^ and CD8^+^ T-cells and cross-present antigens, dendritic cell maturation in tumor tissue is frequently dysregulated, shifting the balance toward immature states [12]. At present, CD1a is commonly accepted as a marker of monocytic [13], and immature DCs [14], whereas CD208 (DC-LAMP) is recognized as a marker of mature DCs [15].

However, the specific roles of DCs subsets and their prognostic significance in CRC progression remain only partially understood and are mostly contradictory. Several studies have reported a positive prognostic impact of CD1a^+^ DCs in CRC [16], while other reports have described the immunosuppressive role of CD1a^+^ DCs and their negative influence on patient survival in CRC [17]. Similarly, CD208^+^ DCs have been associated with a favorable prognosis in CRC in one study [15], whereas other studies have linked it to adverse outcomes [12].

In the gut mucosa, immature DCs constantly sample the environment by taking up antigens from the lumen through phagocytosis and macropinocytosis [18]. Upon maturation and migration to gut-associated lymphoid tissues, these DCs present processed antigens to T-cells, which can then elicit the adaptive immune response towards pathogen clearance or tolerogenic pathways for maintaining homeostasis [19]. However, evidence remains limited on how DCs in non-tumor adjacent mucosa (NAM) may influence the initiation, progression and clinical course of CRC.

It remains unclear whether the spatial localization of DCs has prognostic significance in CRC. High-resolution mapping by Miller et al. revealed a sharp dichotomy between tumor center and invasive margin, with DCs density peaking in invasive margin, where high cell densities predict better survival [20]. Comparative studies assessing DCs distribution and prognostic impact between primary CRC and liver metastases, critical for understanding the loss of immune surveillance in metastases [5], are lacking.

A synthesis of literature has highlighted three major gaps: limited data on quantitative and spatial profiles of CD1a^+^ and CD208^+^ DCs in CRC from NAM through primary tumor to liver metastases; the absence of studies addressing landscape of DCs in synchronous versus metachronous CRC; few, often inconsistent, studies linking these subsets to patient outcomes. The aim of the present study was therefore to map the distribution of CD1a^+^ and CD208^+^ DCs from NAM through pCRC to LM, and to determine how compartment-specific cell counts associate with overall survival in synchronous versus metachronous cases.

## Material and methods

### Patients

All patients who underwent curative resection of pCRC followed by hepatic resection for the first recurrence of CRC at Pilsen University Hospital between 1999 and 2021 were retrospectively reviewed. LM detected at pCRC diagnosis defined the stage IV synchronous cohort (*n* = 80); LM identified 17 (1–59) months (median (interval)) after pCRC resection defined the stage I–III metachronous cohort (*n* = 100).

Inclusion criteria required first-episode LM, curative-intent surgery for both pCRC and LM, complete clinical and survival data, and available good quality formalin-fixed paraffin-embedded (FFPE) tissue. Exclusion criteria were multiple primaries, pre-operative extra-hepatic disease, previous liver resections, neoadjuvant chemoradiotherapy, emergency surgery, or death within 30 days post-operation. Ninety-nine patients fulfilled all criteria (55 with synchronous LM and 44 with metachronous LM).

Demographic, pathological, and clinical variables were extracted (Table S1). Tumors were staged according to the criteria of AJCC 8th edition; most were histological type NOS, grade 2. The cohorts differed only in median LM size (larger in the metachronous group) and in the proportion of patients receiving adjuvant FOLFOX (lower in the metachronous group). The study followed the Declaration of Helsinki (2013) and was approved by the local ethics committee (300/2020, 17 June 2020).

### Pathology and immunohistology

FFPE tissues of pCRC, LM, and NAM from each patient were identified and cut into 4-μm sections. Sample for NAM was taken from the resection margin (oral or aboral), which was the closest to the tumor, with a median distance from the tumor 34 mm (range: 4–200 mm). In case of multiple LMs, we selected the metastatic tumor with the least regressive changes. One or two tissue sections were mounted onto BOND Plus Microscope Slides (Cat#00270, Leica Biosystems Newcastle Ltd., Newcastle, UK). Immunohistochemical detection of CD1a^+^ and CD208^+^ cells was performed using fully automated BOND RXm IHC/ISH stainer. Ready-to-use monoclonal primary antibodies for CD1a (clone MTB1) from Leica Biosystems (Newcastle Ltd., United Kingdom) and CD208 (clone EPR24265-8) from Abcam (Abcam Ltd., United Kingdom) were used. Binding of primary antibodies with their targets was visualized using horseradish peroxidase (HRP)-linker antibody conjugate system (Bond™ Polymer Refine Detection) with a diaminobenzidine as a chromogen. Sections were counterstained with Mayer’s hematoxylin and embedded into Micromount mounting medium (Leica Biosystems Newcastle Ltd., United Kingdom). Appropriate positive (tonsils) and negative tissue control samples were used throughout.

### Image analysis

Whole-slide scans were acquired using an Olympus VS200 scanner (Olympus, Shinjuku, Japan). Regions of interest (ROIs) in pCRC and LM, including tumor center (TC), inner margin (IM), outer margin (OM), and peritumoral zone (PT), were annotated in QuPath v.0.4.3 using custom scripts (https://github.com/sergii01-cuni/script_zones) (Figure S1) and analyzed. ROIs were selected based on the recommendations from the International Immunooncology Biomarkers Working Group [21]. Additionally, tumor invasive margin was divided into inner and outer parts (a 500-µm width each) based on earlier reports by us and others on differential densities of immune cells in those regions in CRC [2, 22], hepatocellular carcinoma [23] and melanoma [22].

NAM was annotated as a single region above the muscularis mucosa, encompassing surface epithelium, intestinal crypts, and lamina propria, with exclusion of dysplasia, crypt lumina, and artifacts. Tumor borders were defined at the interface between malignant nests and adjacent non-tumor tissue, excluding luminal surface, large vessels, normal mucosa, dysplastic epithelium, muscularis propria, supportive stroma > 2 mm, extracellular mucin, fat, necrosis, abscesses, hemorrhages, and artifacts. CD1a^+^ and CD208^+^ cell densities were calculated for NAM and each ROI as the number of immunopositive cell profiles per total ROI area. To eliminate skewness in the distribution, the raw data were converted into percentile values for survival analysis and then categorized into two groups: low (below the 25th percentile) and high (25th–100th percentile).

Data on densities and distribution of CD3^+^, CD8^+^, and CD45RO^+^ T-cells in pCRC and LM in the same cohort of patients were already published by us [2]. In the current study, we invited them to explore relationship between dendritic cells and T-cells.

### Follow-up

Follow-up continued through December 2023. Median surveillance after liver metastasectomy was 84 in the synchronous cohort and 61 months in the metachronous cohort. Patients were reviewed every three months for two years, then semi-annually; each visit included tumor-marker assays, chest X-ray, abdominal ultrasound, and CT. PET or MRI was added at the multidisciplinary team’s discretion.

### Outcomes

The endpoint of the study was overall survival (OS), measured from resection of LM to death from any cause. Patients without death were censored at their last follow-up. OS after LM surgery was not statistically different between groups (data not shown).

### Statistical methods

Continuous variables with non-normal distributions were presented as median (range) and compared using the Mann–Whitney U test or, for repeated measures, Friedman ANOVA with Wilcoxon matched-pairs tests (Bonferroni adjusted). Categorical variables were expressed as counts (%). OS was estimated by Kaplan–Meier analysis and compared with the log-rank test; prognostic value of predictors was assessed by Cox regression with hazard ratios (HRs) for high vs. low groups. Multiple regression with backward stepwise elimination identified predictors of DCs density in ROIs; model quality was assessed by R^2^ and Fisher’s F-test, with elasticity coefficients quantifying covariate influence. Associations between variables were tested by Spearman correlation. Analyses used GraphPad Prism 9.0; survival modeling employed the finalfit package, with significant Cox results visualized in survival and survminer. Two-sided *p* < 0.05 was considered statistically significant.

The schematic representations were created using the BioRender platform (BioRender.com, Toronto, Canada).

## Results

### Morphology and topography of CD1a^+^ and CD208^+^ DCs in NAM, pCRC, and LM

**Fig. 1:**
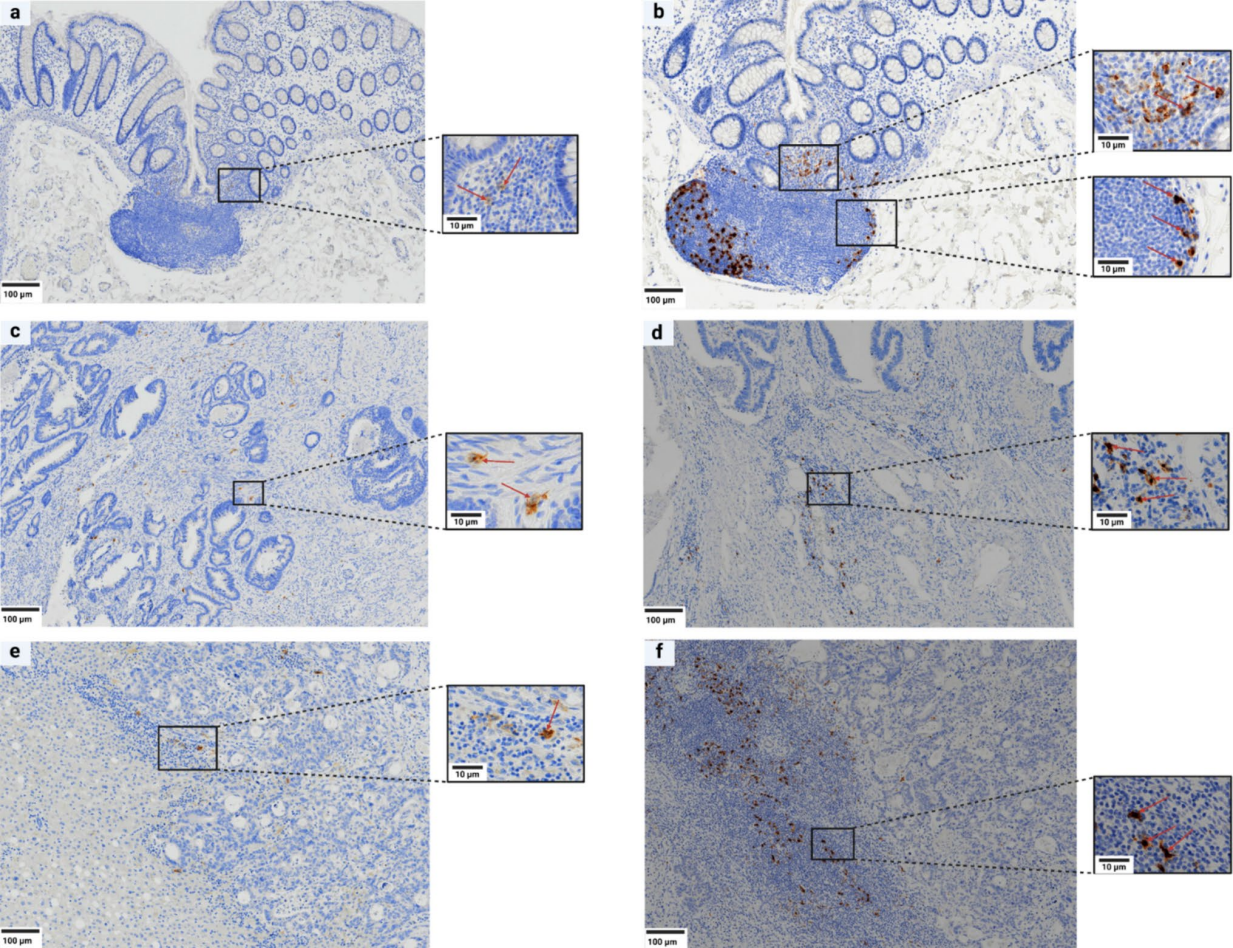
Immunoperoxidase staining for CD1a^+^ and CD208^+^ dendritic cells (DCs) in non-tumor adjacent mucosa (a, b), pCRC (c, d) and LM (e, f). **a** CD1a^+^ DCs are extremely rare in the lamina propria. **b** CD208^+^ DCs, rounded and stellate-shaped and form micro-clusters of approximately 10–20 cells. **b** CD208^+^ DCs accumulate in the mantle and marginal zones of mucosa-associated lymphoid follicles. **c** CD1a^+^ DCs are sparse and predominantly confined to stromal spaces between tumor glands. **d** CD208^+^ DCs are mostly associated with lymphoid aggregates. **e** CD1a^+^ DCs are exceptionally scarce, observed as solitary elements within stromal areas and occasionally within lymphoid aggregates (LA). **f** CD208^+^ DCs are mostly associated with LA DCs: dendritic cells; pCRC: primary colorectal cancer; LM: liver metastases; LA: lymphoid aggregates

Rounded CD1a^+^ DCs were extremely rare in the lamina propria of NAM; in addition, they were occasionally present within the marginal zone of lymphoid follicles (LF) associated with NAM (Fig. 1a). Rounded and stellate-shaped CD208^+^ DCs were larger; they spanned the lamina propria forming micro-clusters of 10–20 cells and also accumulated in mantle and marginal zones of LF (Fig. 1b).

In pCRC, CD1a^+^ cells were sparse, confined to stromal spaces between glands (Fig. 1c) with slightly higher density at the invasive front and sporadically dispersed in PT and OM. CD208^+^ cells were abundant in the stromal regions surrounding the tumor and within lymphoid aggregates (LA) (Fig. 1d), which were more frequent toward the PT region.

In LM, CD1a^+^ DCs were exceptionally scarce, appearing as solitary elements in stroma and LA (Fig. 1e). CD208^+^ DCs were abundant, forming micro-clusters in the TC and IM, accumulating predominantly in the LA, which were, however, more numerous in the OM (Fig. 1f).

TC and IM of LM were also enriched in LA (Figure S2a-d), which were more frequent than in pCRC. Also, LA at the tumor invasive margin was denser, had rounded shapes, and resembled tertiary lymphoid structures (TLS). Collectively, LM thus displays a pronounced imbalance between scant and dispersed immature CD1a^+^ cells and spatially organized clusters of mature CD208^+^ DCs.

### Distribution of CD1a^+^ and CD208^+^ DCs between TC of pCRC and NAM

**Fig. 2:**
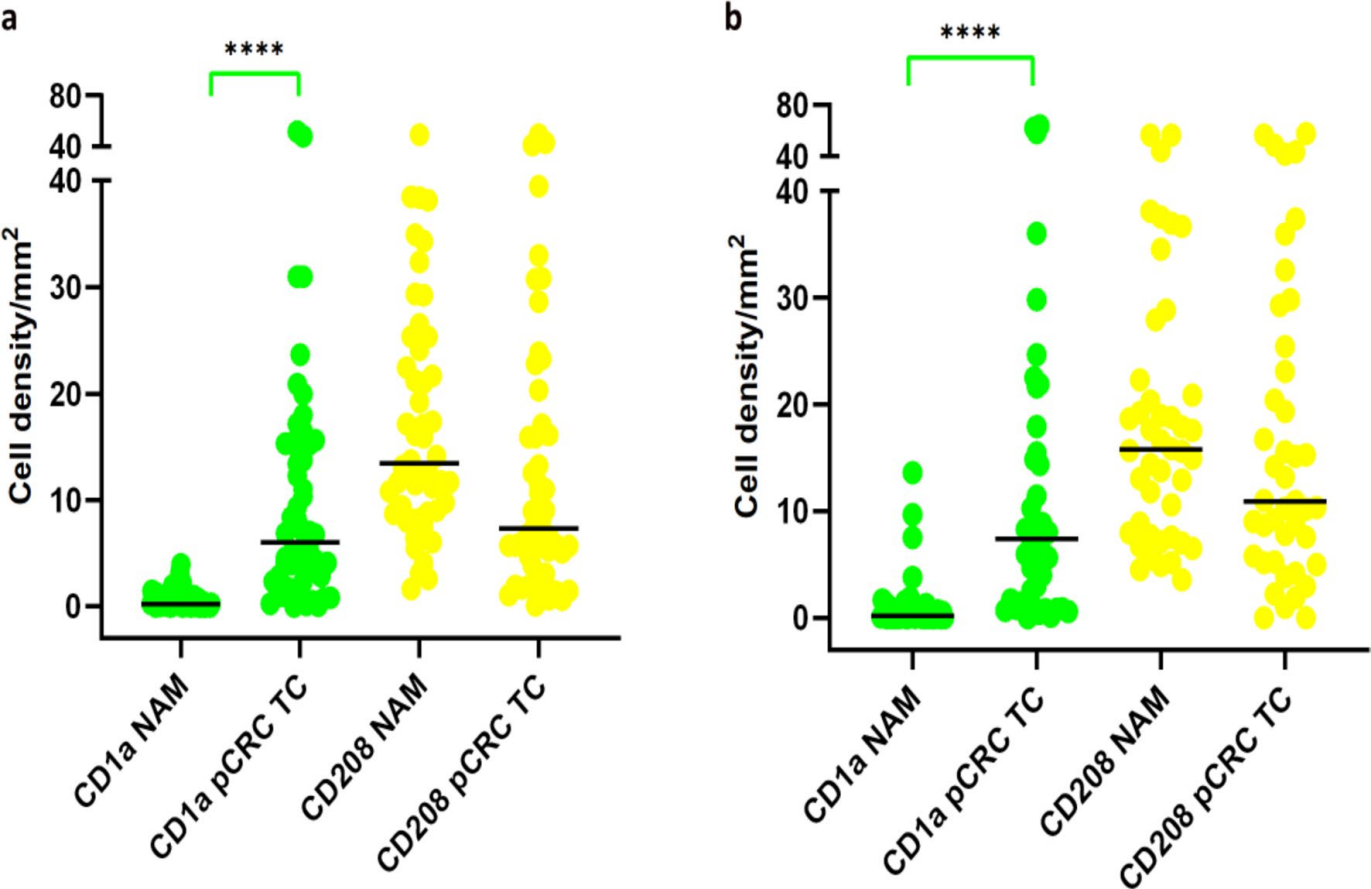
Statistics depicting the spatial distribution of CD1a^+^ and CD208^+^ cells per mm^2^ in NAM and TC of pCRC in patients with synchronous metastasis **a** and metachronous metastasis **b**. Black lines: medians, ****- *p* < 0.0001 Abbreviations: NAM: non-tumor adjacent mucosa, TC: tumor center, pCRC: primary colorectal cancer

Analysis of CD1a^+^ cells in NAM and in the TC of pCRC showed significantly greater cell densities in the TC of pCRC in both cohorts (Fig. 2a, b). By contrast, the density of CD208^+^ DCs was not statistically significant different between the two tissues in either group (Fig. 2a, b).

The number of CD208^+^ cells within NAM was significantly higher than the number of CD1a cells in both patient groups (*p* = 0.0001). No statistically significant difference in densities of either CD1a^+^ or CD208^+^ cells in NAM were observed between groups.

We found significant correlation between densities of CD1a^+^ and CD208^+^ DC in the TC, and between CD1a^+^ DCs in the TC and NAM in the cohort with synchronous metastasis, whereas in patients with metachronous metastasis densities of CD1a^+^ DC and CD208^+^ DC correlated only in the TC (Table S2).

### Distribution of CD1a^+^ and CD208^+^ cells in pCRC and LM

**Fig. 3:**
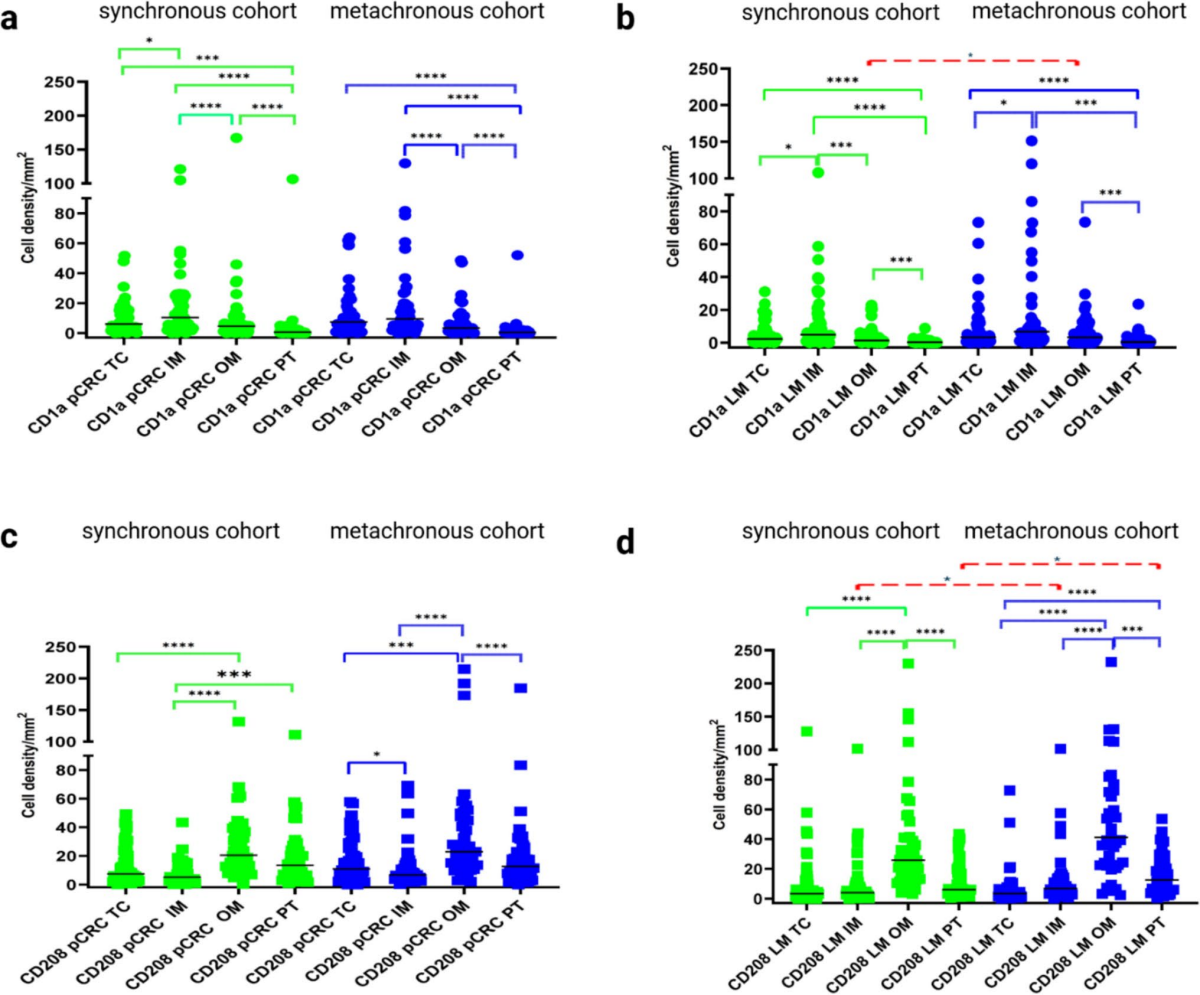
Statistics depicting the spatial distribution of CD1a^+^ (a, b) and CD208^+^ (c, d) cells per mm^2^ in ROIs in pCRC (a, c) and LM (b, d) in patients with synchronous and metachronous LM. Black lines: medians, red lines: significant between synchronous and metachronous cohorts; * *p* < 0.05, ** *p* < 0.01, *** *p* < 0.001, **** *p* < 0.0001. Abbreviations: ROI: region of interesting, LM: liver metastasis, TC: tumor center, IM: inner margin, OM: outer margin, PT: peritumoral zone, pCRC: primary colorectal cancer

In pCRC, CD1a^+^ density was highest at the IM, following the order IM > TC > OM > PT in synchronous cases and IM ≈ TC > OM > PT in metachronous cases (Fig. 3a). In LM, both cohorts showed order IM > TC > OM > PT (Fig. 3b), with greater density in OM of synchronous LM than metachronous LM (*p* < 0.05).

CD208^+^ cells in pCRC displayed order OM > PT = TC ≥ IM in both cohorts, with no intergroup differences; in LM the pattern was OM > PT > TC = IM, but IM and PT of metachronous LM contained more CD208^+^ cells than synchronous LM (both *p* < 0.05) (Fig. 3c, d). CD208^+^ cells predominated over CD1a^+^ in OM and PT of both pCRC and LM in both cohorts (*p* < 0.05), whereas in synchronous disease CD1a^+^ cells exceeded CD208^+^ in pCRC IM (*p* < 0.01) and LM TC (*p* < 0.05).

Compared between pCRC and LM, CD1a^+^ density was higher in every pCRC compartment than in the corresponding compartment of LM in the synchronous cohort (*p* < 0.05), while CD208^+^ density was greater in TC of pCRC in both cohorts (*p* < 0.05).

Spearmanʼs analysis revealed significant correlations for CD1a^+^ and CD208^+^ DCs between all ROIs within pCRC and within LM (Table S3, S4), with the strongest links consistently observed between adjacent ROIs (TC and IM, IM and OM, OM, and PT). In synchronous pCRC, the strongest correlations involved CD1a^+^ cells in TC with CD208^+^ cells in IM and TC, whereas in LM CD1a^+^ cells in TC correlated with CD208^+^ cells in OM. In metachronous pCRC, CD1a^+^ DCs in IM correlated with CD208^+^ cells in PT, IM, and OM, whereas in LM the key associations were between CD1a^+^ cells in TC with CD208^+^ cells in OM and TC. No significant correlations were found between corresponding ROIs of pCRC and LM, except for CD1a^+^ cells in OM (*p* = 0.05) in metachronous cases.

### Survival analysis

**Fig. 4:**
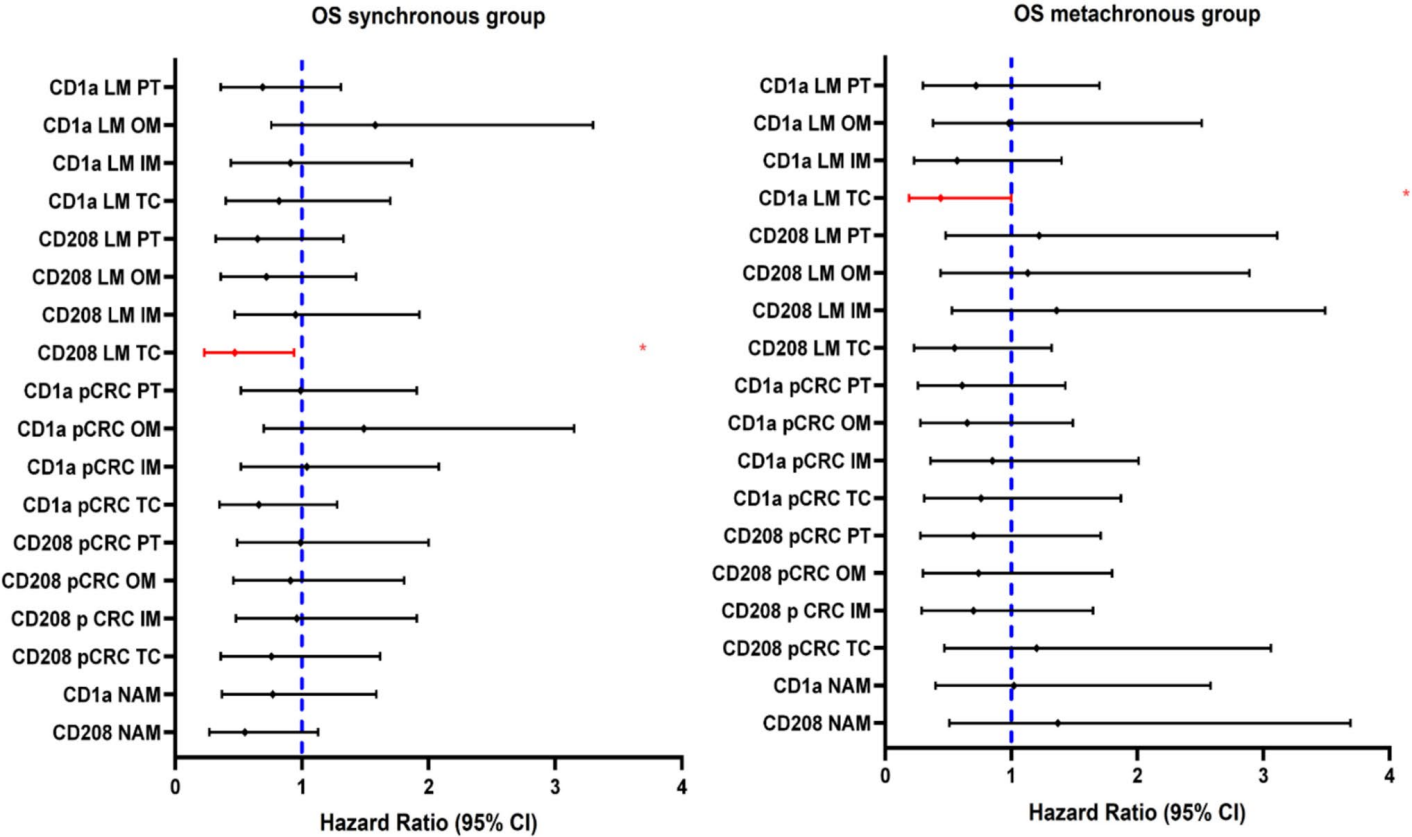
Forest plots of univariable hazard ratios for OS for synchronous and metachronous groups. Information on patient numbers and univariable HR values is provided in Tables S5 and S6. Abbreviations: NAM: non-tumor adjacent mucosa, pCRC: primary colorectal cancer, LM: liver metastasis, TC: tumor center, IM: inner margin, OM: outer margin, PT: peritumoral zone, OS: overall survival. Red line, * – statistically significant variable (*p* < 0.05)

Survival analysis demonstrated that higher CD208^+^ cell density in the TC of synchronous LM and higher CD1a^+^ density in the TC of metachronous LM were associated with reduced mortality risk: HR = 0.47; 95% CI: 0.23–0.94; *p* = 0.033 and HR = 0.44; 95% CI: 0.19–1.00; *p* = 0.050, respectively, although the last association was borderline (Fig. 4, Table S5, S6).

Kaplan–Meier analysis confirmed association of CD208^+^ cells in the TC of synchronous LM with longer OS (Figure S3a) with the curve showing consistently higher survival across the entire observation period. CD1a^+^ cell density in the TC of metachronous LM was significantly associated with a longer OS (Figure S3b).

Based on survival analysis results, multiple linear regression was used to identify independent predictors of CD208^+^ DCs density in TC of synchronous LM and CD1a^+^ DC density in TC of metachronous LM. No valid model was obtained for CD1a, whereas in synchronous LM a robust model identified CD208 in the IM of LM as the strongest predictor (Eq. 1).

1 $${\text{CD}}208{\text{ TC of LM }} = 1.753 + 0.475 \times {\text{CD}}208{\text{ IM of LM}}$$

*R*^2^ = 0.533, *p* < 0.0001.

The model was statistically significant; an increase of 10 CD208^+^ cells in the IM of LM corresponded to a rise of roughly 5 CD208^+^ cells in the TC of LM. Model stability was supported by a high *R*^2^ and a significant *F*-test *p*-value.

Accordingly, we constructed a model predicting density of CD208^+^ DCs in IM of synchronous LM:

2 $$\begin{aligned} {\text{CD}}208{\text{ IM of LM }} = & \, 1.696 \, \times {\text{ CD}}1a{\text{ TC of LM }} - \, 0.180 \, \times {\text{ CD}}1a{\text{ IM of LM }} \\ + \, 2.680 \, \times {\text{ CD}}1a{\text{ PT of LM}} \\ \end{aligned}$$

*R*^2^ = 0.679, *p* < 0.001.

This model was also statistically significant and demonstrated high quality. CD1a^+^ cells in the TC and PT region of LM had a positive effect on density of CD208^+^ cells in the IM of LM, whereas CD1a^+^ cells in the IM of LM had a negative effect.

To quantify the relative impact of each variable, elasticity coefficients were calculated (Table S7). As shown in Table S7, density of CD1a^+^ DCs in the TC of LM had the strongest effect on density of CD208^+^ DCs in the IM of LM.

### Associations of clinical and pathology variables with CD1a^+^ and CD208^+^ DCs

Tables S8–S10 summarize associations between clinicopathological variables and CD1a^+^, CD208^+^ DCs densities across NAM, pCRC, and LM, as well as DCs and chemotherapy timeline and regimens. Statistically significant differences are indicated in the legends for Tables S8–S10.

### Associations between DCs and T-cells

Densities of CD208^+^ DCs in each ROI from NAM to LM significantly correlated with densities of CD3^+^ T-cells in both synchronous and metachronous groups (Table S11, Table S12). Similarly, we found a correlation between CD208^+^ DCs and CD8^+^ T-cells in all ROI of both synchronous and metachronous LM and additionally in TC and IM of pCRC in synchronous group and PT of pCRC in metachronous group. CD208^+^ DCs and CD45RO^+^ T-cells correlated in all tumor ROIs except IM of synchronous LM.

Densities of CD1a^+^ DCs correlated with densities of CD3^+^ T-cells in all ROI but PT of synchronous LM and all ROI but TC of metachronous LM, and additionally in TC and PT of pCRC in synchronous group. CD1a^+^ DCs correlated with CD8^+^ T-cells in TC and IM of synchronous LM and OM of metachronous LM, and additionally in PT of pCRC in synchronous group. CD1a^+^ DCs and CD45RO^+^ T-cells correlated in IM and OM of LM in both groups, and additionally in PT of LM and PT of pCRC in metachronous group and TC of synchronous LM.

## Discussion

The immune landscape of pCRC and its metastases constitutes a dynamic system in which DCs is one the main actors of the immune response. Most previous studies have investigated DCs primarily in pCRC, often as a pooled population restricted to the tumor core, without considering their maturation state or compartment-specific localization. Studies focusing on liver metastases are relatively few and provide only limited insight into DCs heterogeneity [24]. To our knowledge, this is the first study to comprehensively characterize CD1a^+^ and CD208^+^ DCs distribution across NAM and several anatomical compartments of both primary CRC and paired synchronous or metachronous LM, and to directly relate these spatial patterns to patient survival.

Establishing a baseline profile of DCs subsets in NAM could serve as a reference for assessing tumor-associated alterations. We observed high densities of CD208^+^ DCs with an almost complete absence of CD1a^+^ DCs within NAM, yielding a CD208/CD1a ratio of approximately 25:1. Mature CD208^+^ DCs in NAM were predominantly located within mucosa-associated LF, where DCs complete functional maturation and participate in antigen presentation [25]. The absence of correlation between CD208^+^ DCs in NAM and CD1a^+^ or CD208^+^ cells across different ROIs of pCRC suggest their autonomous resident profile in NAM, which is supported by the literature [18].

Greater densities of CD1a^+^ DCs in TC of pCRC vs NAM can reflect CCL2-dependent influx of circulating monocytes that differentiate into moDCs within the TME [18]. Additionally, CD1a^+^ dendritic cells can migrate from the tumor exterior toward tumor interior along chemokines gradients to increase pool of immature DCs there [26]. Similar mechanism can be responsible for higher densities of CD1a^+^ DCs in the tumor interior of LM vs tumor exterior. Across pCRC and LM tissue in both synchronous and metachronous cohorts of patients, CD1a^+^ density was greater in the TC/IM, whereas CD208^+^ density was greater in OM/PT. This pattern is consistent with the mechanisms described by Jie Chen, Yuhang Duan et al., in which hypoxic stress in the tumor core hampers dendritic cell maturation and sustains their immature state [27]. LA, particularly those exhibiting features of TLS serve as a niche for terminal DCs maturation [28, 29]. Abundance of LA in the tumor exterior vs tumor interior in our and other studies [28, 30] can drive the migration of immature DCs along outward CCL19/CCL21 gradients [31], and their accumulation and maturation within LA in peritumoral compartments (OM, PT) [28, 29]. This interpretation is supported by correlation analysis, which revealed the strongest associations between CD1a^+^ cells in TC and CD208^+^ cells in OM in both pCRC and LM. Although current study was not aimed to characterize TLS, which would require more extensive phenotyping, we observed numerous mature DCs within peripheral zones of LA, which is one of distinguishing features of TLS vs. simple LA [32].

Compared between pCRC and LM, greater densities of CD1a^+^ DCs were observed in corresponding compartments of pCRC in synchronous disease. Synchronous LM establish a VEGF-A/IL-10/TGF-*β*-rich tolerogenic niche [4] that can suppress CCR7-mediated homing and maturation of DCs precursors [33], preventing their accumulation in the metastatic nodule. Circulating monocytes are instead retained at the CD1a^+^ stage by GM-CSF, CXCL8, and CCL2 gradients in the primary tumor [34, 35], turning the pCRC into a functional “monocyte reservoir”. This distribution is consistent with the concomitant immunity model [2], whereby the primary tumor serves as the main antigen source, partially restrains growth of secondary lesions, and limits influx of antigen-presenting cells into metastases. Also, greater CD208^+^ cell density was found in the TC of pCRC compared with LM in both cohorts. This finding suggests that the metastatic niche is less permissive for DCs maturation [36]. Kupffer cells release a broad spectrum of immunosuppressive and tumor-promoting mediators, including IL-10, TGF-*β*, IL-6, VEGF, and MMPs, which collectively inhibit dendritic-cell differentiation and antigen presentation while fostering tumor cell invasion, proliferation, and angiogenesis [37], which may explain the impaired accumulation of mature DCs in LM.

We also observed a distinct immune architecture between synchronous and metachronous LM: in metachronous LM, CD1a^+^ cell density was higher in OM and CD208^+^ cell density was higher in IM and PT than in synchronous lesions (*p* < 0.05). Miyagawa 2004 reported higher numbers of mature DCs in metachronous over synchronous LM [38]. As reported by Wenchao Xu et al., after primary tumor resection, metastases evolve under prolonged cytokine–chemokine stimulation, where sustained low levels of GM-CSF, CXCL8, and CCL2 maintain monocyte influx, progressively expanding the CD1a^+^ pool [39].

**Fig. 5:**
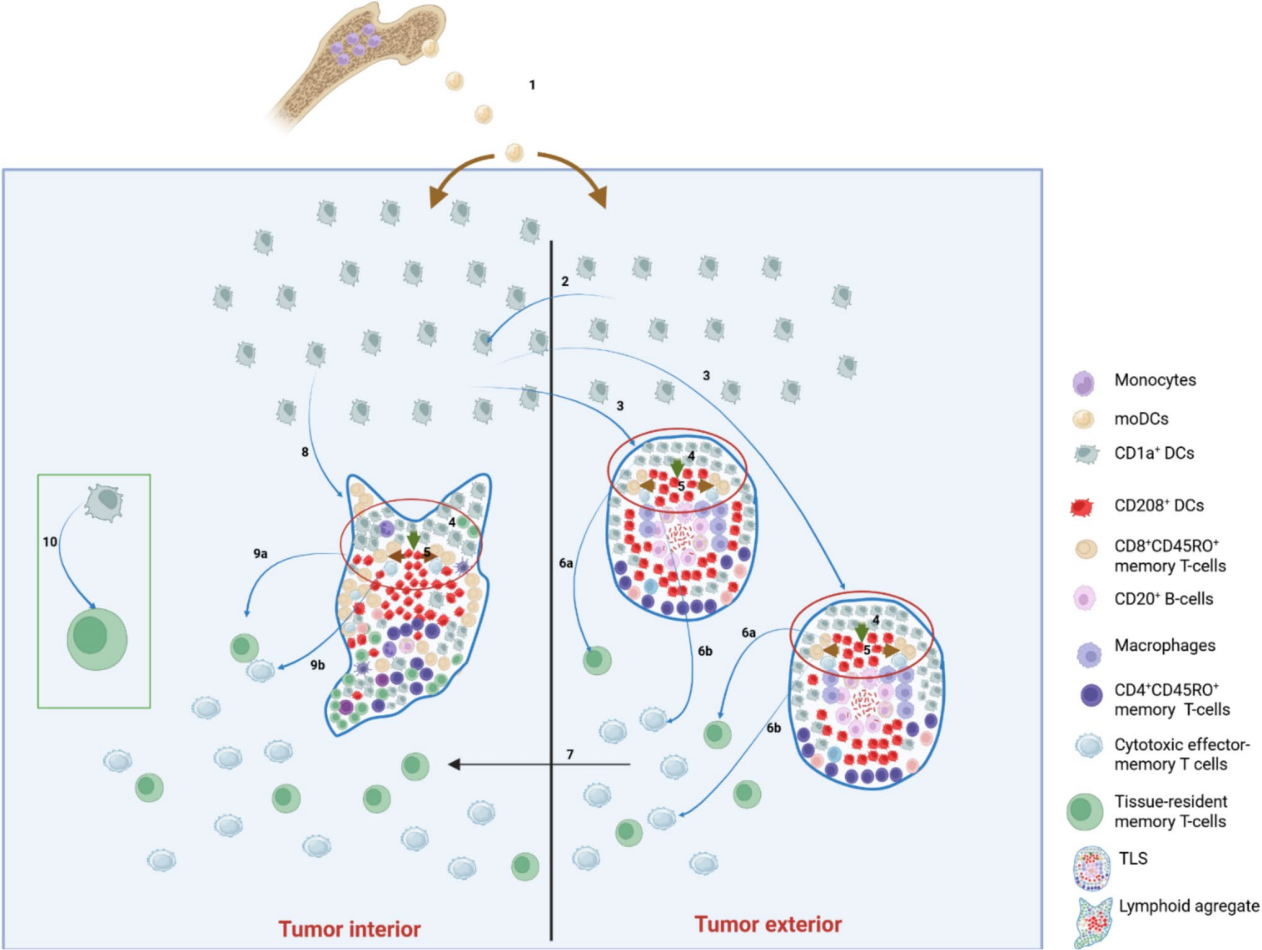
Suggested mechanisms of antitumor effects of dendritic cells in CRC LM. Monocytes differentiate into monocyte-derived dendritic cells (moDCs) [12], which enter the tissue and form a pool of CD1a^+^ dendritic cells in both tumor interior (TC + IM) and tumor exterior (OM + PT) (1) [12]. CD1a^+^ dendritic cells from the tumor exterior migrate toward the tumor interior along chemokines gradients to increase pool of immature DCs there (2) [34]. In the tumor interior CD1a^+^ dendritic cells capture tumor antigens and then migrate along CCL19/CCL21 gradients back toward numerous tertiary lymphoid structures (TLS) in the tumor exterior (3) [31]. In TLS these partially activated CD1a^+^ cells complete maturation into CD208^+^ dendritic cells (4) [28, 29], which present antigens to CD4⁺CD45RO^+^ and CD8^+^CD45RO^+^ memory T-cells (5) [28, 29]. Memory T-cells proliferate, differentiate into cytotoxic effector-memory (6a) and tissue-resident memory T-cells (6b), migrate into the tumor interior (7) [28, 29], where they execute antitumor effector functions and sustain the local adaptive immune response. This mechanism is ubiquitous for synchronous and metachronous LM. Simultaneously, maturation of DCs occurs within the tumor interior, forming CD208^+^ clusters frequently surrounded by CD4^+^CD45RO^+^ and CD8^+^CD45RO^+^ memory T-cells within lymphoid aggregates [41], which also serve to local antigen presentation but may lack a follicular organization (8) [41]. Following antigen presentation by mature CD208^+^ dendritic cells and activation of CD4^+^CD45RO^+^ and CD8^+^CD45RO^+^ T-lymphocyte, memory T-cells proliferate and differentiate into cytotoxic effector-memory and tissue-resident memory T-cells which infiltrate the tumor tissue (9a, b) [41]. This mechanism may explain favorable survival associations of mature DCs in TC of synchronous LM. In addition to the mechanisms described above, CD1a^+^ dendritic cells can activate tissue-resident memory T-cells within tumor stroma (10). Metachronous LM are characterized by greater densities of CD45RO^+^ memory T-cells [2] and CD1a^+^ immature DCs, therefore in metachronous LM DCs have better chances to activate memory T-cells, including their activation within tumor stroma under conditions of minimal co-stimulation. This mechanism may explain favorable survival associations of immature DCs in TC of metachronous LM. CRC: colorectal cancer; LM: liver metastases; DCs: dendritic cells; TC: tumor center; IM: inner margin; OM: outer margin; PT: peritumoral zone; TLS: tertiary lymphoid structures, LA: lymphoid aggregates

The prognostic impact of DCs subsets in CRC has been widely debated, with previous studies reporting conflicting results and providing little information on synchronous versus metachronous liver metastases. We demonstrate here that a high density of mature CD208^+^ cells in the TC of synchronous LM was associated with nearly a two-fold reduction in the risk of death. In our cohort, LA enriched in mature CD208^+^ DCs were frequently observed in TC and IM of LM (Fig. 2S). Several recent studies confirmed that TLS can develop in the center of metastases [40]. We also observed from strong to moderate correlation between CD208^+^ DCs and CD8^+^ and CD45RO^+^ T-cells in TC and IM of LM, which point on cooperation of mature dendritic cells with cytotoxic and memory T-cells. Therefore, despite predominance of TLS and mature DCs in tumor exterior, intratumoral LA seem to be superior as local hubs for T-cell recruitment, antigen presentation, clonal expansion, generation of effector-memory T-cells and tissue-resident memory T-cells, which infiltrate then the tumor tissue [41], as shown on Fig. 5. All these mechanisms collectively can contribute to a stronger anti-tumor immune response associated with improved overall survival [32]. Our theory is supported by other authors, who demonstrated that compared with peritumoral TLS, intratumoral LA had a stronger antitumor effect [29, 30]. Multiple regression models revealed CD1a^+^ DCs in the TC and PT region as the strongest predictors for densities of CD208^+^ cells in the tumor interior of synchronous LM. Therefore, CD1a^+^ cells in the PT region can play a central role in establishing the population of mature DCs in the tumor center of synchronous liver metastases, as evidenced by the high elasticity coefficient. This result corroborates the above concept of recruitment of immature DCs into the tumor and underscores the importance of rapid maturation of CD1a^+^ cells within intratumoral LA. We can hypothesize that presence and functional integrity of LA within core of synchronous LM, where CD1a^+^ maturate into CD208^+^ cells, governs the clinical impact of DCs.

In metachronous CRC, a higher density of CD1a^+^ DCs in the TC of LM was associated with improved OS. Besides greater densities of immature DCs, metachronous LM in our cohort also harbor more CD45RO^+^ memory T-cells [2], which play a role in long-term anti-tumor immunity and the prevention of cancer recurrence [42]. It has also been shown that immature DCs are capable of activating memory T-cells under conditions of minimal co-stimulation, reflecting a fundamental distinction between the activation mechanisms of naïve lymphocytes and memory cells [43]. Therefore, in metachronous LM immature DCs have better chances to activate memory T-cells (Fig. 5). Furthermore, IFN-*γ* released by memory T-cells can directly induce rapid functional maturation of dendritic cells, enhancing their antigen-presenting capacity [44]. Eventual interaction between CD1a^+^ immature DCs and CD45RO^+^ T-cells in metachronous LM is supported by correlation analysis in our study.

When thoroughly validated, the present findings imply several clinical applications. First, lower counts of mature DCs in synchronous LM and immature DCs in metachronous LM may identify a group of patients at risk of relapse, who would require thorough monitoring after resection of LM. Second, they could guide immunotherapy strategies. Indeed, several studies demonstrated that tumors with higher intratumor mature DCs are more likely to respond to immune checkpoint blockade even in mismatch repair-proficient tumors [45]. Moreover, strategies aimed at achieving maturation of DCs in situ [46] or DCs-based vaccination [47] can be suggested.

DCs markers were quantitatively assessed on digitized whole-slide images using specialized software, minimizing observer bias. To our knowledge, this is the first study to compare the compartment-specific prognostic significance of immature CD1a⁺ and mature CD208^+^ DCs using triplicate samples of NAM, pCRC, and synchronous or metachronous LM, capturing the key maturation axis relevant to antigen presentation. Immune profiling of NAM alongside pCRC offers insights into TME evolution, highlights TC as a critical immune regulatory compartment, and reveals favorable prognostic associations of these DCs subsets in LM, which may inform risk stratification and immunomodulatory strategies.

However, several limitations should be noted. Relatively small sample size and the lack of comprehensive molecular and mutational data in some patients can limit the power to detect certain survival associations. While the low occurrence of MSI-high tumors may restrict the broader applicability of our results, we are confident in their relevance to our patients, all of whom developed LM, because the prevalence of MSI-high status is low in CRC with distant metastases [48]. Expression of immune checkpoint molecules was not tested in the current study, however; none of patients in the cohort was treated with immune checkpoint blockers due to absolute prevalence of MSI-low tumors. Heterogeneity of adjuvant regimens precluded a reliable assessment of interactions between therapy and the immune system. Also, to obtain a comprehensive understanding of the spatial distribution of DCs within pCRC and LM tissues, as well as to evaluate DCs migration across different compartments, future studies should employ spatial transcriptomics in combination with DCs tracking experiments. Finally, although CD1a/CD208 immunophenotyping captured the most relevant DCs maturation dynamics in this study, we did not assess other myeloid populations that may participate in interactions between the tumor and the immune system, which should be addressed in future studies.

## Conclusions

This study provides the first comprehensive spatial mapping of CD1a^+^ and CD208^+^ DCs from NAM through pCRC to paired LM. We identified opposing gradients of CD1a^+^ and CD208^+^ DCs consistent with an recruitment-maturation axis, with immature CD1a⁺ cells enriched in TC and mature CD208^+^ cells predominating in tumor exterior. Importantly, survival analysis revealed distinct prognostic patterns depending on the temporal pattern of metastasis: in synchronous LM, higher CD208^+^ density in the TC was associated with improved OS, whereas in metachronous LM, favorable outcomes were linked to higher CD1a^+^ density in the TC. These findings indicate that patient prognosis depends not only on abundance of DCs but also on their distribution, with the TC being a key compartment, and on the metastatic timeline. Taken together, our results highlight the clinical relevance of spatial immune profiling of DCs as a potential tool for prognostic assessment and therapeutic stratification in CRC patients with liver metastases.

## Supplementary Information

Below is the link to the electronic supplementary material.

Supplementary file1 (DOCX 77 kb)

Supplementary file2 (PDF 1729 kb)

Supplementary file3 (DOCX 8417 kb)

Supplementary file4 (DOCX 102 kb)

## Data Availability

All data generated or analyzed during this study are included in this article and its additional material files. Further enquiries can be directed to the corresponding author.

## Abbreviations

**CRC**: Colorectal cancer
**DCs**: Dendritic cells
**FFPE**: Formalin fixed-paraffin embedded tissue
**HRs**: Hazard ratios
**IM**: Inner margin
**LA**: Lymphoid aggregates
**LF**: Lymphoid follicles
**LM**: Liver metastasis
**NAM**: Non-tumor adjacent mucosa
**OM**: Outer margin
**OS**: Overall survival
**pCRC**: Primary colorectal cancer
**PT**: Peritumoral zone
**ROIs**: Regions of interests
**TC**: Tumor center
**TLS**: Tertiary lymphoid structures
**TME**: Tumor microenvironment
