# Supplementary material for: Spatial patterns and prognostic relevance of CD1a+ immature and CD208+ mature dendritic cells in colorectal cancer from non-tumor adjacent mucosa to liver metastases
Source: Cancer Immunol Immunother. 2025 Dec 18;75(1):4. doi: 10.1007/s00262-025-04238-2 (PMC12714677; doi:10.1007/s00262-025-04238-2)
Supplement: Supplementary file 2 — Supplementary file2 (PDF 1729 kb) [file 262_2025_4238_MOESM2_ESM.pdf]

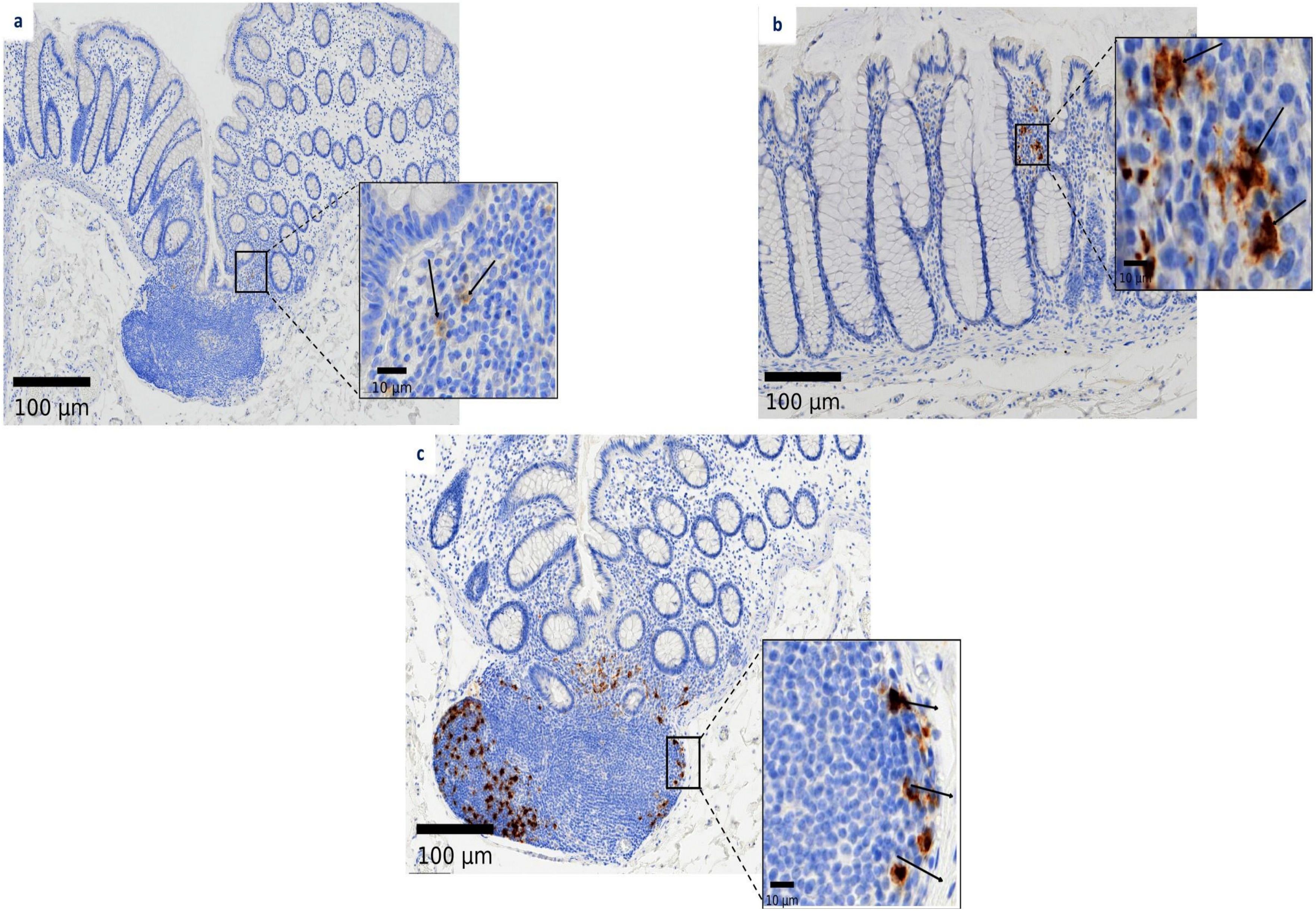

Figure S1. Immunoperoxidase staining for CD1a⁺ and CD208⁺ dendritic cells (DCs) in non-tumor adjacent normal mucosa. (**a**) CD1a⁺ DCs are extremely rare in the lamina propria. (**b**) CD208⁺ DCs, rounded and stellate-shaped and form micro-clusters of approximately 10–20 cells. (**c**) CD208⁺ DCs accumulate in the mantle and marginal zones of mucosa-associated lymphoid follicles. You need to position the small rectangle differently in a. b, c

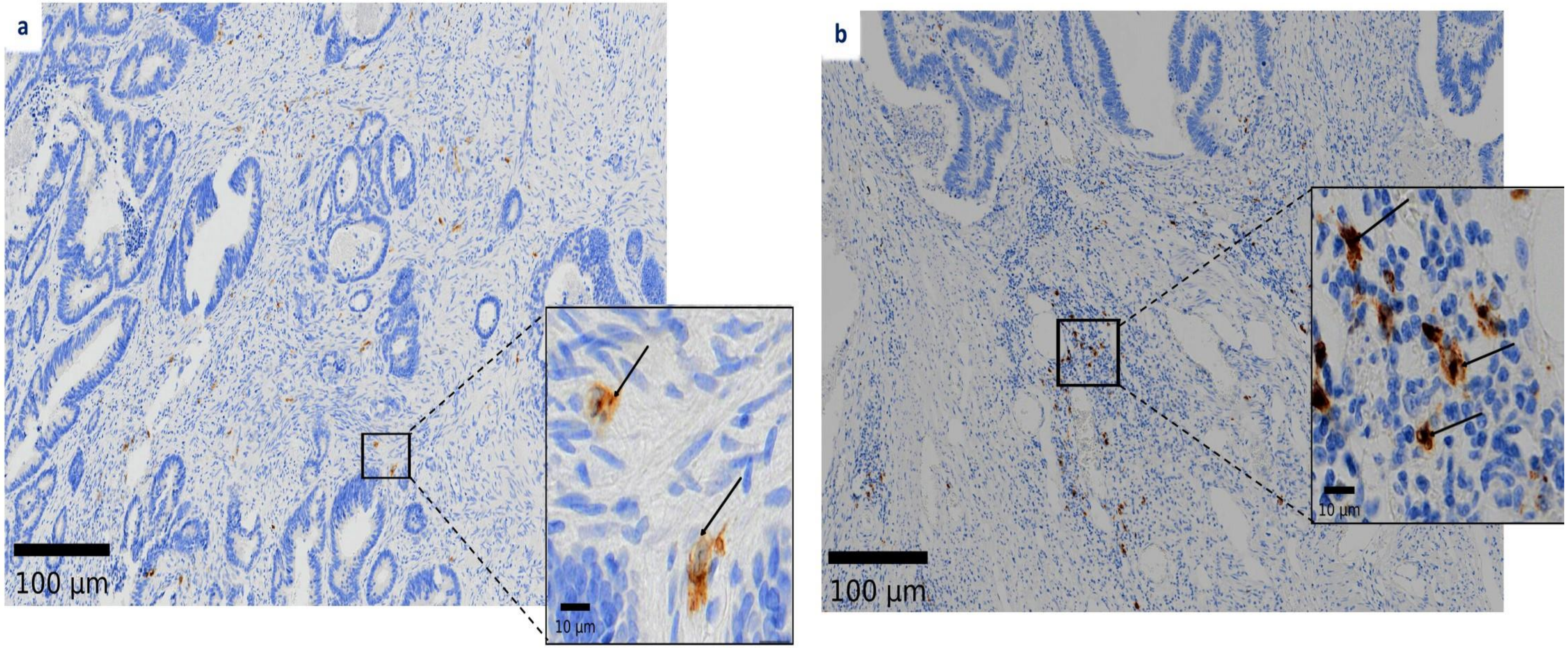

Figure S2. Immunoperoxidase staining for CD1a$^+$ and CD208$^+$ dendritic cells (DCs) in primary colorectal cancer. (**a**) CD1a$^+$ DCs are sparse and predominantly confined to stromal spaces between tumor glands. (**b**) CD208$^+$ DCs are mostly associated with lymphoid aggregates.

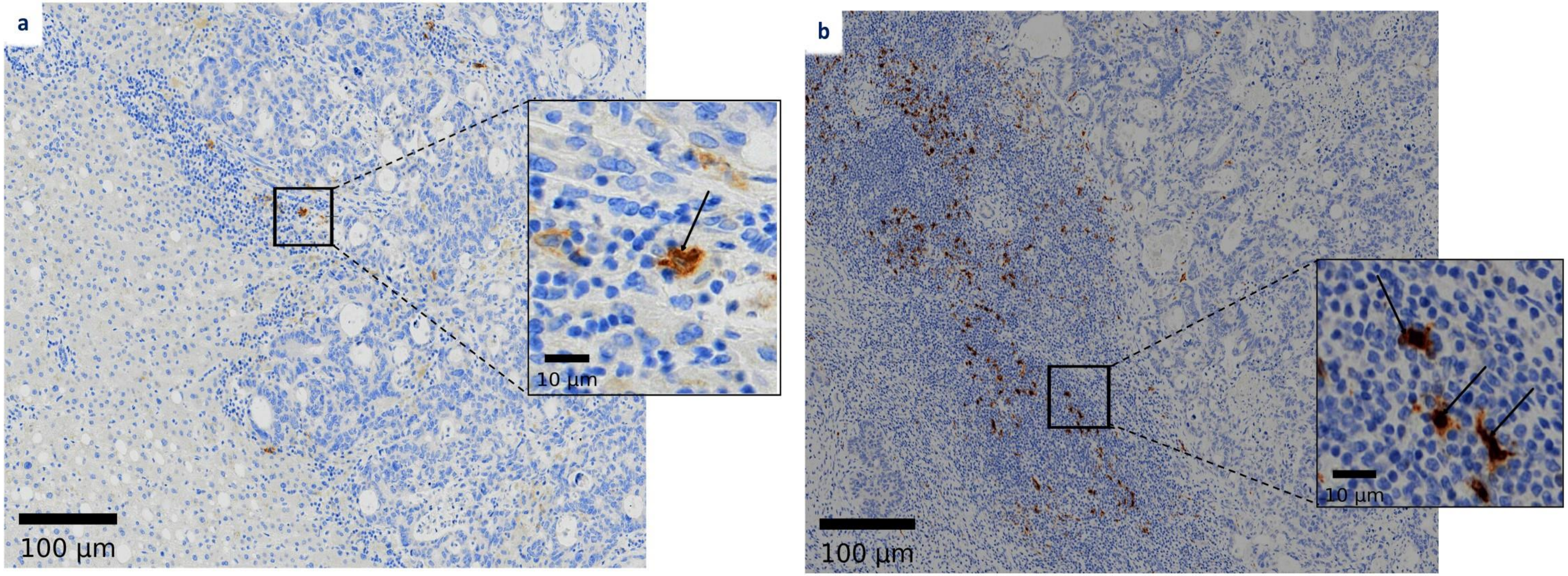

Figure S3. Immunoperoxidase staining for $CD1a^{+}$ and $CD208^{+}$ dendritic cells (DCs) in colorectal liver metastases. (**a**) $CD1a^{+}$ DCs are exceptionally scarce, observed as solitary elements within stromal areas and occasionally within lymphoid aggregates (LA). (**b**) $CD208^{+}$ DCs are mostly associated with LA. b

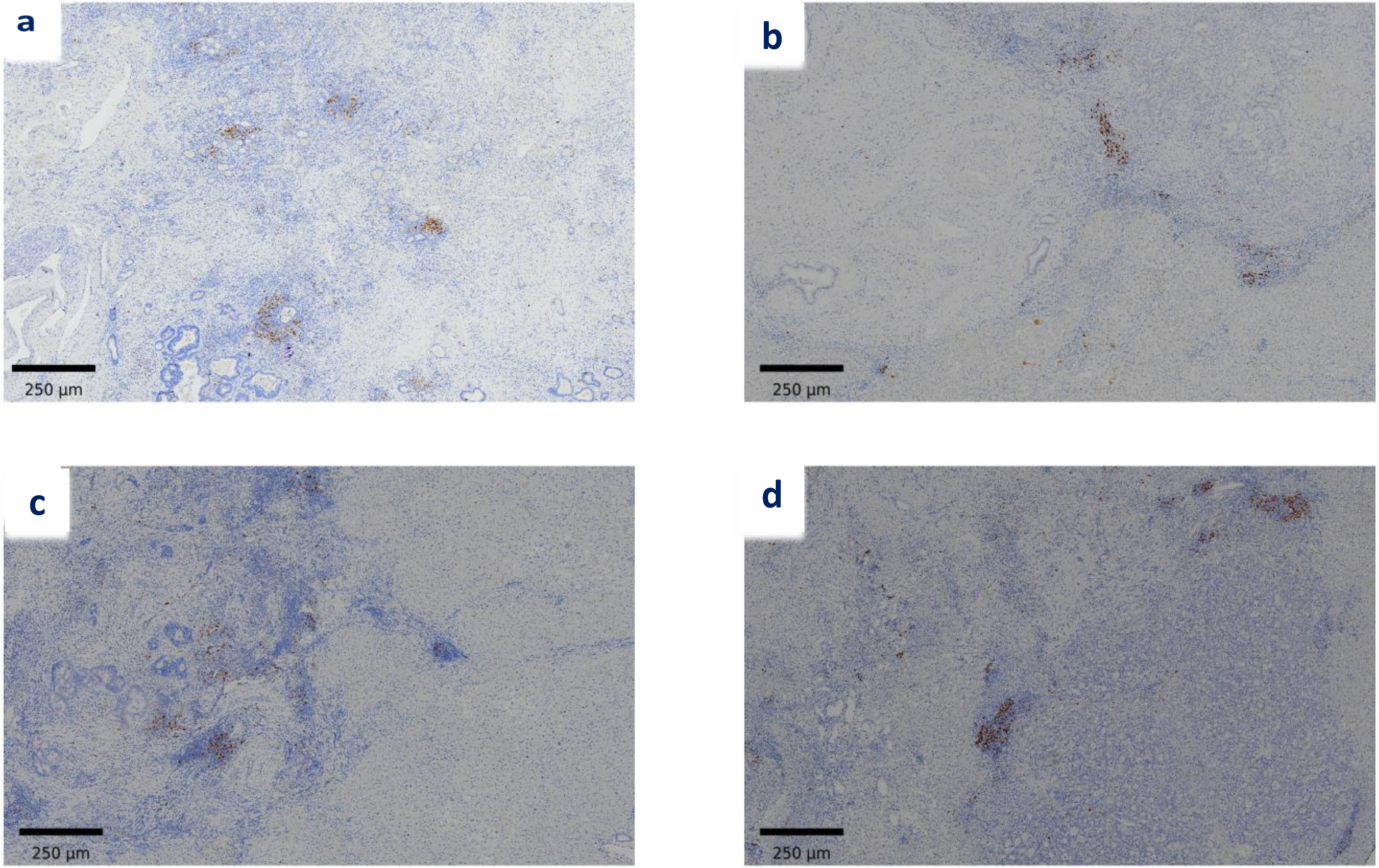

Figure S4. Lymphoid aggregates containing CD208⁺ dendritic cells in colorectal liver metastases. (**a**) Tumor center, (**b-d**) tumor invasive margin.

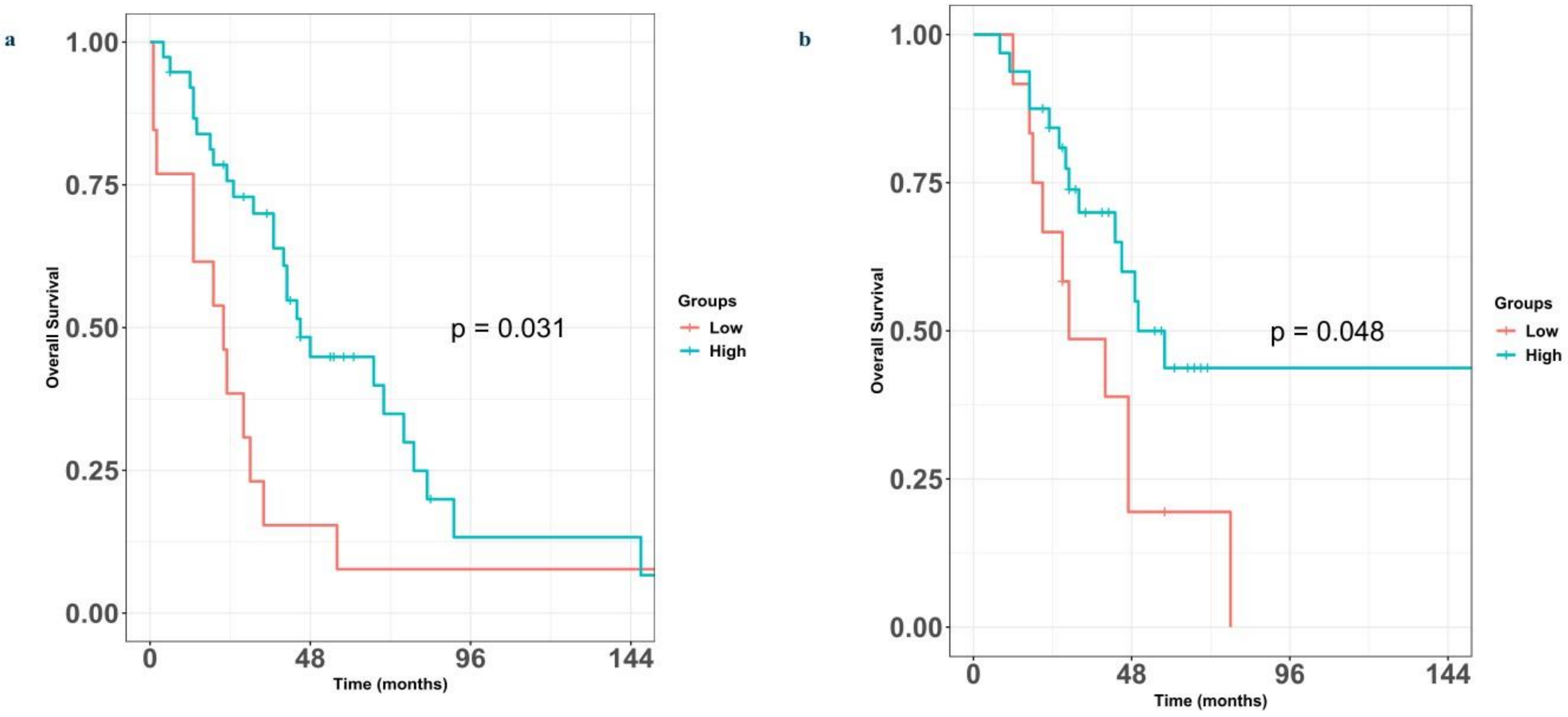

Figure S5. Kaplan–Meier survival curves comparing patients with high versus low densities of CD1a or CD208 positive dendritic cells.
a —OS in the synchronous metastasis cohort, stratified by $CD208^{+}$ cells in LM TC.
b — OS in the metachronous metastasis cohort, stratified by $CD1a^{+}$ cells in LM TC.
Two-sided log rank p values are reported on each graph.
Abbreviations: OS – overall survival, LM: liver metastasis, TC: tumour center.
