# Supplementary material for: Spatial patterns and prognostic relevance of CD1a+ immature and CD208+ mature dendritic cells in colorectal cancer from non-tumor adjacent mucosa to liver metastases
Source: Cancer Immunol Immunother. 2025 Dec 18;75(1):4. doi: 10.1007/s00262-025-04238-2 (PMC12714677; doi:10.1007/s00262-025-04238-2)
Supplement: Supplementary file 3 — Supplementary file3 (DOCX 8417 kb) [file 262_2025_4238_MOESM3_ESM.docx]

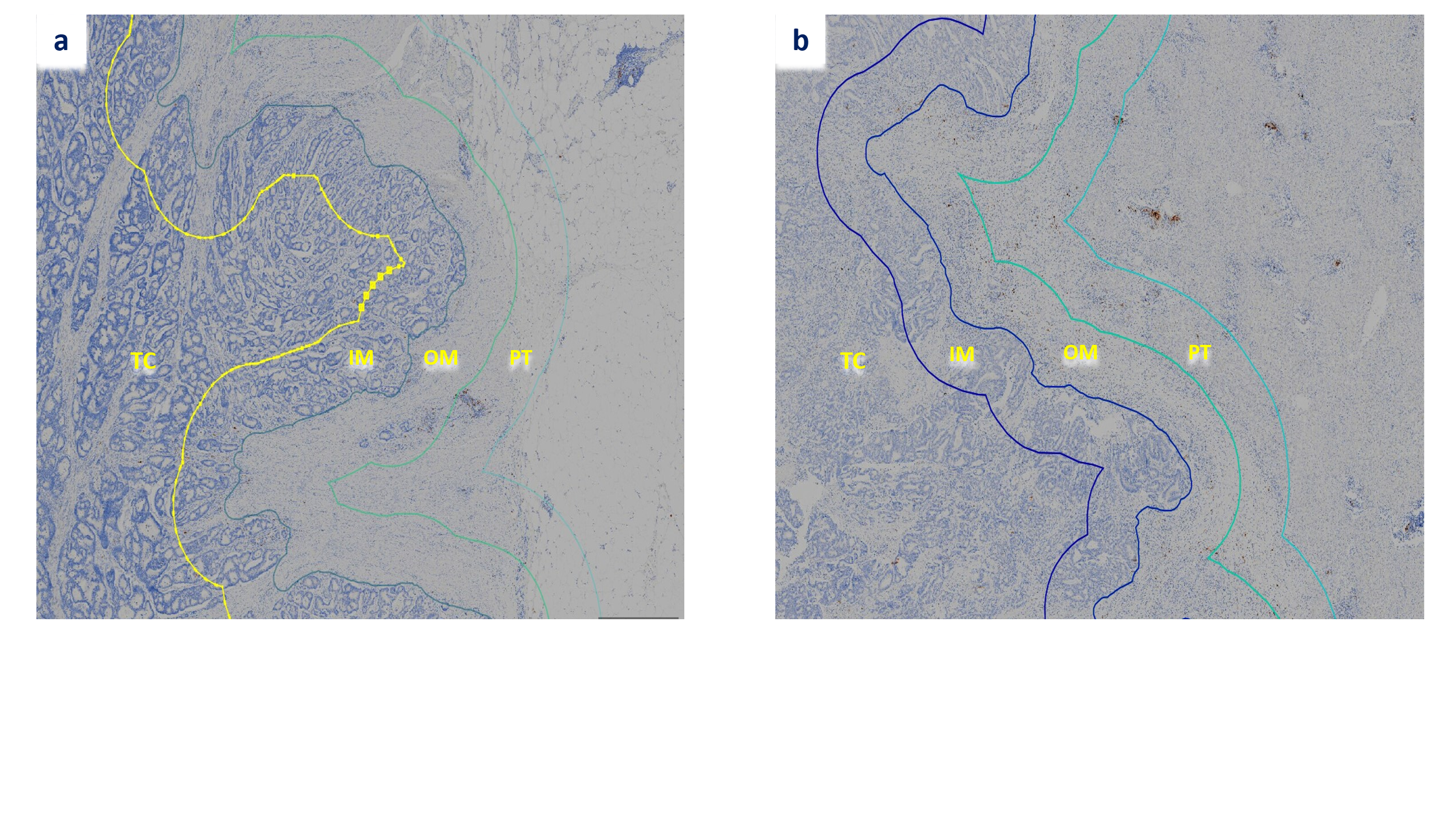


**Figure S1.** Regions of interest in primary colorectal cancer (a) and liver metastasis (b) of colorectal cancer annotated on whole-slide images after anti-CD208 staining using QuPath software. Tumor center (TC), and inner margin (IM), outer margin (OM), and peritumoral zone (PT), a 500-µm width each.


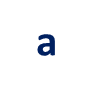

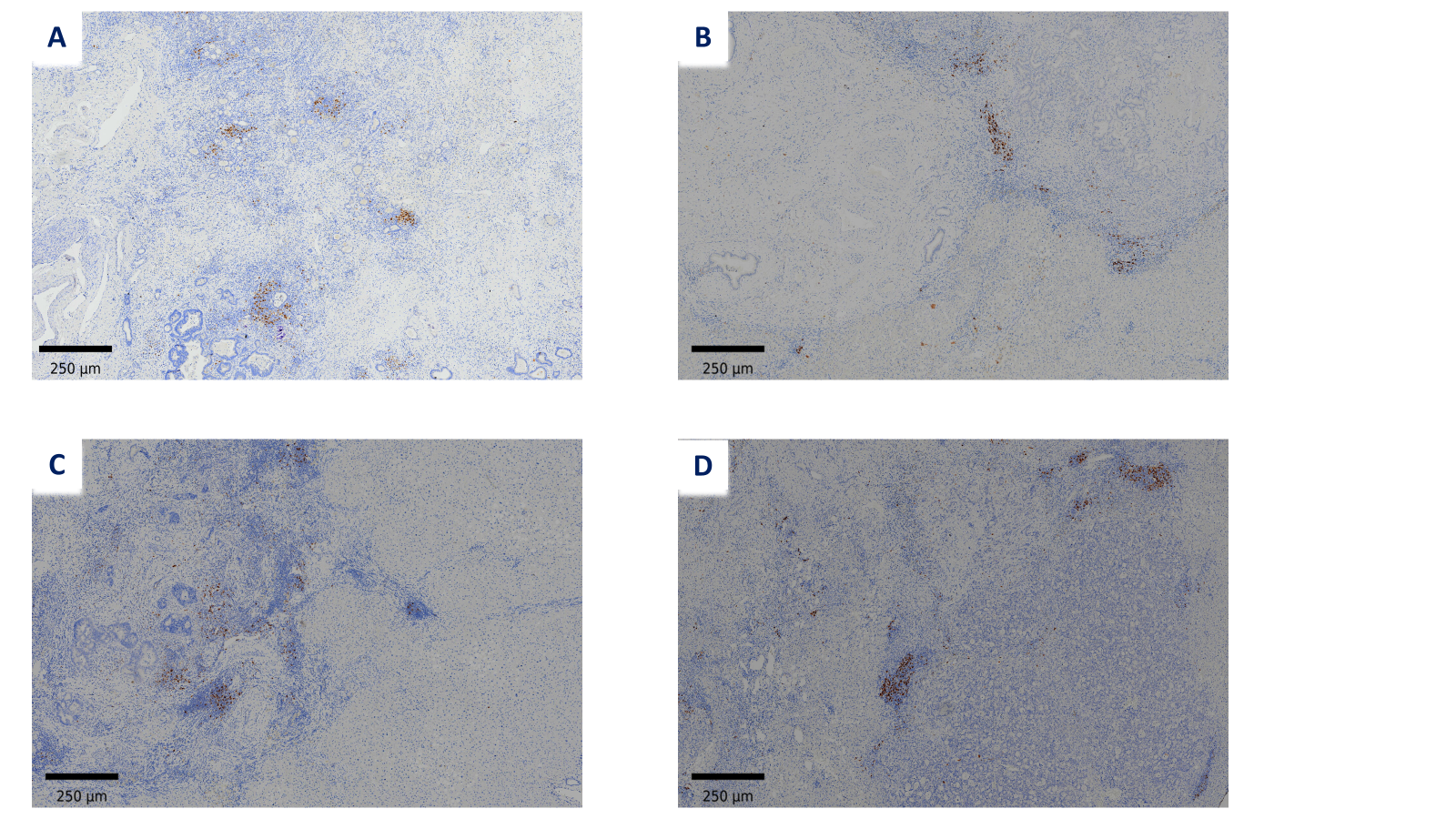


**b**

**d**

**c**

**Figure S2.** Lymphoid aggregates containing CD208⁺ dendritic cells in colorectal cancer liver metastases. (**a**) tumor center, (**b-d**) tumor invasive margin.


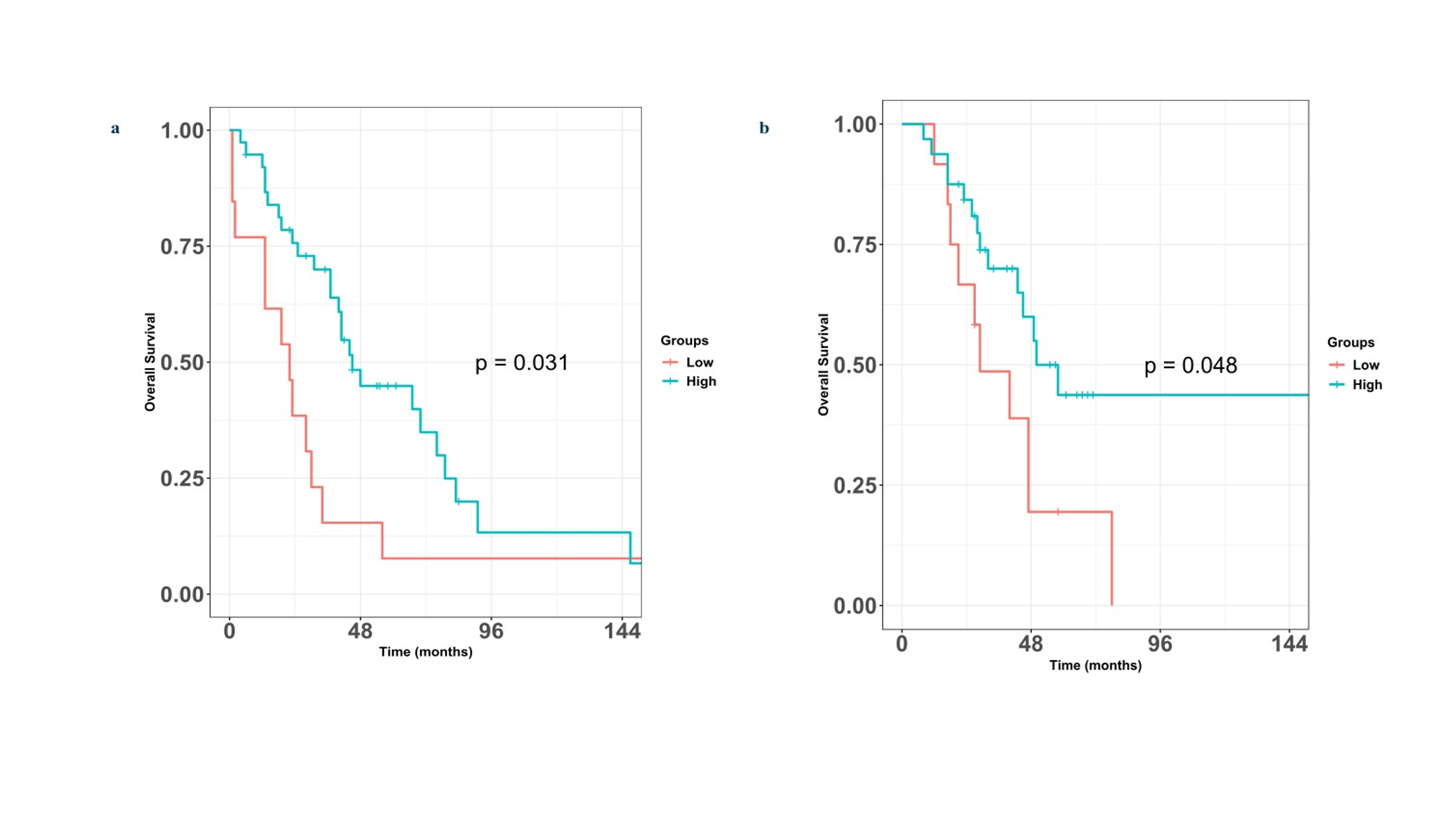


**Figure S3.** Kaplan–Meier survival curves comparing patients with high versus low densities of CD1a^+^ or CD208^+^ dendritic cells.

a — OS in the synchronous metastasis cohort, stratified by CD208⁺ cells in LM TC (38 and 13 patients in High and Low groups, respectively).

b — OS in the metachronous metastasis cohort, stratified by CD1a⁺ cells in LM TC (39 and 13 patients in High and Low groups, respectively).

Two-sided log rank p values are reported on each graph.

Abbreviations: OS: overall survival, LM: liver metastasis, TC: tumor center.
