# Supplementary material for: Spatial patterns and prognostic relevance of CD1a+ immature and CD208+ mature dendritic cells in colorectal cancer from non-tumor adjacent mucosa to liver metastases
Source: Cancer Immunol Immunother. 2025 Dec 18;75(1):4. doi: 10.1007/s00262-025-04238-2 (PMC12714677; doi:10.1007/s00262-025-04238-2)
Supplement: Supplementary file 4 — Supplementary file4 (DOCX 102 kb) [file 262_2025_4238_MOESM4_ESM.docx]

**Supplementary tables**

**Table S1.** Clinical backgrounds of enrolled patients and histopathological features of primary and metastatic tumours

| Parameter | | Synchronous, N=55 | Metachronous, N=44 | P value |
| --- | --- | --- | --- | --- |
| Age at the diagnosis (years), median (min-max) | | 62 (29-78) | 64 (46-73) | 0.31 |
| Gender | male | 34 (61.8%) | 29 (65.9%) | 0.67 |
|  | female | 21 (38.2%) | 15 (34.1%) |  |
| **Primary tumour** | |  | | |
| Location | Right colon | 12 (21.8%) | 8 (18.2%) | 0.65 |
|  | Left colon | 43 (78.2%) | 36 (81.8%) |  |
| Size (cm), median (min-max) | | 4.3 (1.0-7.5) | 3.5 (0.8-8.3) | 0.055 |
| Pathologic T stage | T1  T2  T3  T4 | 0 (0.0%)  1 (1.8%)  48 (87.3%)  6 (10.9%) | 1 (2.3%)  6 (13.6%)  35 (79.5%)  2 (4.5%) | 0.25 |
| Histological type | NOS  Mucinous  Other | 51 (92.7%)  2 (3.6%)  2 (3.6%) | 39 (88.6%)  4 (9.1%)  1 (2.3%) | 0.27 |
| Pathologic N stage | N0 | 13(23.6%) | 13(29.5%) | 0.51 |
|  | N1 | 22 (40.0%) | 16 (36.4%) |  |
|  | N2 | 20 (36.4%) | 15 (34.1%) |  |
| AJCC 8th staging | Stage I  Stage II  Stage III  Stage IV | 55 (100%) | 1 (2.3%)  12 (27.3%)  31 (70.4%) |  |
| Grade | 1  2  3 | 12 (21.8%)  37 (67.3%)  6 (10.9%) | 14 (31.8%)  26 (59.1%)  4 (9.1%) | 0.77 |
| KRAS status | mutated | 20 (36.4%) | 11 (25.0%) | 0.26 |
|  | WT | 27 (49.1%) | 25 (56.8%) |  |
|  | not tested | 8 (14.5%) | 8 (18.2%) |  |
| BRAF status | mutated | 0 (0.0%) | 1 (2.3%) | 0.27 |
|  | WT | 44 (80.0%) | 35 (79.5%) |  |
|  | not tested | 11 (20.0%) | 8 (18.2%) |  |
| MMR status | deficient | 1 (1.8%) | 1 (2.3%) | 0.94 |
|  | proficient | 28 (50.9%) | 25 (56.8%) |  |
|  | not tested | 26 (47.3%) | 18 (40.9%) |  |
| MSI status | high | 1 (1.8%) | 1 (2.3%) | 0.9 |
|  | low | 42 (76.4%) | 35 (79.5%) |  |
|  | not tested | 12 (21.8%) | 8 (2.3%) |  |
| CEA ng/ml | | 6.8 (1.0-945.0) | 8.9 (0.8-1649.0) | 0.90 |
| CEA | <5ng/mL | 18 (46.2%) | 13 (46.4%) |  |
|  | >5ng/mL | 21 (53.8%) | 15 (53.6%) | 0.98 |
| Number of examined lymph nodes | | 13 (1-34) | 11 (0-43) | 0.38 |
| Lymph node ratio, median (25-75perc) | | 0.22 (0-0.57) | 0.13 (0-0.32) | 0.14 |

**Table S1 (continued).** Clinical backgrounds of enrolled patients and histopathological features of primary and metastatic tumors

| Parameter | | Synchronous, N=55 | Metachronous, N=44 | P value |
| --- | --- | --- | --- | --- |
| **Liver metastases** | |  |  |  |
| Number, median (min-max) | | 2 (1-24) | 1 (1-7) | 0.08 |
| Size (cm), median (min-max) | | 2.0 (0.4-26.0) | 2.7 (0.6-7.1) | **0.0180** |
| Grade | 1  2 | 21 (39.6%)  32 (60.4%) | 18 (42.9%)  24 (57.1%) | 0.7580 |
| Metastasis resection  R status | R0  R1 | 40 (76.9%)  12 (23.1%) | 29 (69.0%)  13 (31.0%) | 0.3980 |
| Chemotherapy ± biological regimens | CHT alone  CHT + anti-VEGF  CHT + anti-EGFR  no treatment  not known | 28 (50.9%)  11 (20.0%)  9 (16.4%)  4(7.3%)  3 (5.4%) | 30 (68.2%)  4 (9.1%)  5 (11.4%)  2 (4.5%)  3 (6.8%) | 0.07 |
| Chemotherapy regimens | FOLFOX  dle Gramont  FOLFIRI  others | 31 (56.4%)  7 (12.7%)  4 (7.3%)  6 (10.9%) | 16 (36.4%)  9 (20.5%)  2 (4.5%)  12 (27.3%) | **0.048** |
| Chemotherapy ± biological therapy timing | before liver surgery  after liver surgery  no | 37 (67.3%)  12 (21.8%)  6 (10.9%) | 11 (25.0%)  32 (72.7%)  1 (2.3%) | **0.0001** |
| Response to preoperative therapy | CR-PR  PD-SD | 22 (59.5%)  15 (40.5%) | 4 (40%)  6 (60%) | 0.27 |
| Response to postoperative  therapy | CR-PR  PD-SD | 1 (8.3%)  11 (91.7%) | 5 (16.1%)  26 (83.9%) | 0.5 |

Notes: Bold values indicate statistical significance at the p < 0.05 level.

Abbreviations: NOS: not otherwise specified; AJCC: the American joint committee on cancer; CEA: the carcinoembryonic antigen; CHT: chemotherapy; CR: complete response; PR: partial response; SD: stable disease; PD: progressive disease; MMR: mismatch repair; MSI: microsatellite instability.

**Table S2** Spearman’s rank correlation between CD208^+^ and CD1a^+^ cells in NAM and TC of pCRC

|  | CD208 NAM | CD208 pCRC TC | CD1a NAM |
| --- | --- | --- | --- |
| Synchronous group | | | |
| CD208 pCRC TC | -0.02 |  |  |
| CD1a NAM | 0.21 | 0.17 |  |
| CD1a pCRC TC | 0.1 | 0.38** | 0.32* |
| Metachronous group | | | |
| CD208 pCRC TC | -0.01 |  |  |
| CD1a NAM | 0.13 | -0.02 |  |
| CD1a pCRC TC | 0.06 | 0.40** | 0.11 |

Abbreviations: NAM: non tumor adjacent mucosa, pCRC: primary colorectal cancer; TC: tumor center.

* p<0.05; ** p<0.01

**Table S3** Spearman’s rank correlation between CD208^+^ and CD1a^+^ cell densities across individual ROIs in pCRC.

|  | **CD208**  **pCRC TC** | **CD208**  **pCRC  IM** | **CD208**  **pCRC  OM** | **CD208**  **pCRC PT** | **CD1a**  **pCRC TC** | **CD1a**  **pCRC IM** | **CD1a**  **pCRC OM** |
| --- | --- | --- | --- | --- | --- | --- | --- |
| **Synchronous metastasis** | | | | | | | |
| **CD208 pCRC  IM** | 0.68**** |  |  |  |  |  |  |
| **CD208 pCRC  OM** | 0.43*** | 0.59**** |  |  |  |  |  |
| **CD208 pCRC PT** | 0.32** | 0.44*** | 0.63**** |  |  |  |  |
| **CD1a pCRC TC** | 0.38** | 0.48*** | 0.26 | 0.20 |  |  |  |
| **CD1a pCRC IM** | 0.16 | 0.35** | 0.35** | 0.13 | 0.80**** |  |  |
| **CD1a pCRC OM** | 0.06 | 0.20 | 0.34** | 0.20 | 0.63**** | 0.85**** |  |
| **CD1a pCRC PT** | 0.08 | 0.26 | 0.27 | 0.33** | 0.47**** | 0.54**** | 0.61**** |
| **Metachronous metastasis** | | | | | | | |
| **CD208 pCRC  IM** | 0.63**** |  |  |  |  |  |  |
| **CD208 pCRC  OM** | 0.55**** | 0.78**** |  |  |  |  |  |
| **CD208 pCRC PT** | 0.33** | 0.46** | 0.61**** |  |  |  |  |
| **CD1a pCRC TC** | 0.40* | 0.40**** | 0.40** | 0.38** |  |  |  |
| **CD1a pCRC IM** | 0.37* | 0.49*** | 0.49*** | 0.49*** | 0.80**** |  |  |
| **CD1a pCRC OM** | 0.36* | 0.41** | 0.45** | 0.35** | 0.78**** | 0.86**** |  |
| **CD1a pCRC PT** | 0.19 | 0.23 | 0.35** | 0.41** | 0.55**** | 0.63**** | 0.58**** |

Abbreviations: pCRC: primary colorectal cancer; TC: tumor center; IM: inner margin; OM: outer margin; PT: peritumoral zone; ROI: regions of interests

* Spearman’s p<0.05; ** Spearman’s p<0.01, *** Spearman’s p<0.001, **** Spearman’s p<0.0001

**Table S4** Spearman’s rank correlation between CD208‑positive and CD1a‑positive cell densities across individual ROIs in LM.

|  | **CD208**  **LM TC** | **CD208**  **LM IM** | **CD208**  **LM OM** | **CD208**  **LM PT** | **CD1a**  **LM TC** | **CD1a**  **LM IM** | **CD1a**  **LM OM** |
| --- | --- | --- | --- | --- | --- | --- | --- |
| **Synchronous metastasis** | | | | | | | |
| **CD208 LM IM** | 0.62**** |  |  |  |  |  |  |
| **CD208 LM OM** | 0.40** | 0.49*** |  |  |  |  |  |
| **CD208 LM PT** | 0.15 | 0.15 | 0.57**** |  |  |  |  |
| **CD1a LM TC** | 0.48*** | 0.52**** | 0.58**** | 0.34* |  |  |  |
| **CD1a LM IM** | 0.25 | 0.37** | 0.49*** | 0.22 | 0.83**** |  |  |
| **CD1a LM OM** | 0.09 | 0.37** | 0.54**** | 0.35** | 0.53**** | 0.68**** |  |
| **CD1a LM PT** | 0.20 | 0.26 | 0.21 | 0.39** | 0.29 | 0.37** | 0.43*** |
| **Metachronous metastasis** | | | | | | | |
| **CD208 LM IM** | 0.63**** |  |  |  |  |  |  |
| **CD208 LM OM** | 0.45** | 0.68**** |  |  |  |  |  |
| **CD208 LM PT** | 0.30* | 0.33* | 0.35* |  |  |  |  |
| **CD1a LM TC** | 0.51*** | 0.42** | 0.56**** | 0.34* |  |  |  |
| **CD1a LM IM** | 0.18 | 0.35* | 0.54*** | 0.30* | 0.80**** |  |  |
| **CD1a LM OM** | 0.10 | 0.22 | 0.48*** | 0.42** | 0.67**** | 0.84**** |  |
| **CD1a LM PT** | 0.20 | 0.22 | 0.40** | 0.53*** | 0.51*** | 0.56**** | 0.78**** |

Abbreviations: LM: liver metastasis, TC: tumor center, IM: inner margin, OM: outer margin, PT: peritumoral zone, ROI: regions of interests

* p<0.05; ** p<0.01, *** p<0.001, **** p<0.0001

**Table S5.** Hazard ratios for OS between high versus low DCs density per individual ROI of LM in CRC patients with synchronous metastases, corresponding to Figure 4.

| **Marker, region** | **Number (%) of patients in high group** | **HR (95% confidence interval), p value** |
| --- | --- | --- |
| CD1a LM PT | 35 (66.0) | 0.69 (0.36-1.31), p=0.257 |
| CD1a LM OM | 40 (75.5) | 1.58 (0.76-3.30), p=0.221 |
| CD1a LM IM | 39 (75.0) | 0.91 (0.44-1.87), p=0.802 |
| CD1a LM TC | 39 (75.0) | 0.82 (0.40-1.70), p=0.596 |
| CD208 LM PT | 40 (75.5) | 0.65 (0.32-1.33), p=0.239 |
| CD208 LM OM | 40 (75.5) | 0.72 (0.36-1.4)3, p=0.350 |
| CD208 LM IM | 39 (75.0) | 0.95 (0.47-1.93), p=0.888 |
| CD208 LM TC | 38 (74.5) | 0.47 (0.23-0.94), p=0.033 |
| CD1a pCRC PT | 38 (70.4) | 0.99 (0.52-1.91), p=0.981 |
| CD1a pCRC OM | 40 (74.1) | 1.49 (0.70-3.15), p=0.297 |
| CD1a pCRC IM | 40 (74.1) | 1.04 (0.52-2.08), p=0.918 |
| CD1a pCRC TC | 40 (74.1) | 0.66 (0.35-1.28), p=0.218 |
| CD208 pCRC PT | 40 (74.1) | 0.99 (0.49-2.00), p=0.971 |
| CD208 pCRC OM | 40 (74.1) | 0.91 (0.46-1.81), p=0.792 |
| CD208 pCRC IM | 40 (74.1) | 0.96 (0.48-1.91, p=0.903 |
| CD208 pCRC TC | 40 (74.1) | 0.76 (0.36-1.62), p=0.479 |
| CD1a NM | 40 (75.5) | 0.77 (0.37-1.59), p=0.471 |
| CD208 NM | 40 (74.1) | 0.55 (0.27-1.13), p=0.103 |

Abbreviations: HR: hazard ratios; NAM: non-tumor adjacent mucosa; pCRC: primary colorectal cancer, LM: liver metastasis, TC: tumor center, IM: inner margin, OM: outer margin, PT: peritumoral zone

**Table S6.** Hazard ratios for OS between high versus low DCs density per individual ROI of LM in CRC patients with metachronous metastases, corresponding to Figure 4.

| **Marker, region** | **Number (%) of patients in high group** | **HR (95% confidence interval), p value** |
| --- | --- | --- |
| CD1a LM PT | 31 (72.1) | 0.72 (0.30-1.70), p=0.450 |
| CD1a LM OM | 32 (74.4) | 0.98 (0.38-2.51), p=0.972 |
| CD1a LM IM | 32 (74.4) | 0.57 (0.23-1.40), p=0.222 |
| CD1a LM TC | 32 (72.7) | 0.44 (0.19-1.00), p=0.051 |
| CD208 LM PT | 32 (74.4) | 1.22 (0.48-3.11), p=0.676 |
| CD208 LM OM | 32 (74.4) | 1.13 (0.44-2.89), p=0.803 |
| CD208 LM IM | 32 (74.4) | 1.36 (0.53-3.49), p=0.516 |
| CD208 LM TC | 32 (76.2) | 0.55 (0.23-1.32), p=0.179 |
| CD1a pCRC PT | 28 (66.7) | 0.61 (0.26-1.43), p=0.253 |
| CD1a pCRC OM | 31 (70.5) | 0.65 (0.28-1.49), p=0.306 |
| CD1a pCRC IM | 31 (70.5) | 0.85 (0.36-2.01), p=0.717 |
| CD1a pCRC TC | 31 (72.1) | 0.76 (0.31-1.87), p=0.550 |
| CD208 pCRC PT | 32 (74.4) | 0.70 (0.28-1.71), p=0.430 |
| CD208 pCRC OM | 32 (74.4) | 0.74 (0.30-1.80), p=0.503 |
| CD208 pCRC IM | 32 (74.4) | 0.70 (0.29-1.65, p=0.411) |
| CD208 pCRC TC | 32 (74.4) | 1.20 (0.47-3.06, p=0.695) |
| CD1a NAM | 32 (72.7) | 1.02 (0.40-2.58, p=0.972) |
| CD208 NAM | 33 (75.0) | 1.37 (0.51-3.69, p=0.539) |

Abbreviations: HR: hazard ratios; NAM: non-tumor adjacent mucosa; pCRC: primary colorectal cancer, LM: liver metastasis, TC: tumor center, IM: inner margin, OM: outer margin, PT: peritumoral zone

**Table S7.** Elasticity coefficients for Model 1

|  | Coefficient in the model | Average | Elasticity Coefficient |
| --- | --- | --- | --- |
| CD208 LM IM |  | 6.05 |  |
| CD1a LM TC | 1.696 | 2.91 | 0.8158 |
| CD1a LM IM | -0.180 | 7.32 | -0.2178 |
| CD1a LM PT | 2.680 | 0.49 | 0.2171 |

Abbreviations: LM: liver metastasis, TC: tumor center, IM: inner margin, OM: outer margin, PT: peritumoral zone

**Table S8.** Association between CD1a^+^ and Cd208^+^ DCs and clinical and pathological variables in CRC patients with synchronous and metachronous metastases

| **Variables** | **Cell type**  **and location** | **Synchronous** | | | | | **Metachronous** | | | | |
| --- | --- | --- | --- | --- | --- | --- | --- | --- | --- | --- | --- |
|  |  |  | **TC** | **IM** | **OM** | **PT** |  | **TC** | **IM** | **OM** | **PT** |
| Males | **CD1a NAM** | 0 (0-4) |  |  |  |  | 0 (0-10) |  |  |  |  |
|  | CD1a pCRC |  | 7 (0-48) | 13 (0-121) | 5 (0-96) | 0 (0-9) |  | 8 (0-36) | 9 (0-82) | 3 (0-47) | 0 (0-4) |
|  | Cd1a LM |  | 2 (0-24) | 5 (0-108) | 1 (0-23) | 0 (0-3) |  | 2 (0-39) | 6 (0-120) | 2 (0-73) | 0 (0-23) |
|  | CD208 NAM | 13 (3-49) |  |  |  |  | 14 (5-56) |  |  |  |  |
|  | CD208 pCRC |  | 8 (0-43) | 7 (0-25) | 24 (5 -132) | 14 (1-111) |  | 10 (0-57) | 7 (2-50) | 20 (3-115) | 12 (0-83) |
|  | CD208 LM |  | 3 (0-128) | 4 (0-103) | 21 (3-230) | 6 (1-40) |  | 3 (0-21) | 7 (0-102) | 39 (2-131) | 11 (1-45) |
| Females | **CD1a NAM** | 0 (0-3) |  |  |  |  | 0 (0-14) |  |  |  |  |
|  | CD1a pCRC |  | 5 (1-52) | 9 (0-105) | 4 (0-167) | 1 (0-106) |  | 5 (0-64) | 10 (0-130) | 5 (0-49) | 1 (0-52) |
|  | CD1a LM |  | 2 (0-31) | 7 (0-40) | 2 (0-9) | 0 (0-9) |  | 4 (0-73) | 10 (2-151) | 5 (0-30) | 1 (0-7) |
|  | CD208 NAM | 14 (2-114) |  |  |  |  | 19 (4-57) |  |  |  |  |
|  | CD208 pCRC |  | 6 (1-49) | 4 (0-43) | 15 (0-61) | 9 (2-46) |  | 16 (0-58) | 8 (0-69) | 27 (2-192) | 18 (2-185) |
|  | CD208 LM |  | 3 (0-44) | 5 (0-41) | 30 (6-155) | 7 (1-43) |  | 3 (1-73) | 10 (3-58) | 51 (22-232) | 18 (2-54) |
| Age,  above median | CD1a NAM | 0 (0-3)* |  |  | . |  | 0 (0-14) |  |  |  |  |
|  | CD1a pCRC |  | 5 (0-52)* | 6 (0-105) | 2 (0-167) | 0 (0-106) |  | 7 (0-64) | 7 (0-130) | 3 (0-47) | 0 (0-2) |
|  | CD1a LM |  | 2 (0-31) | 5 (0-40) | 1 (0-23) | 0 (0-9) |  | 2 (1-39) | 6 (0-120) | 2 (0-73) | 0 (0-23) |
|  | CD208 NAM  CD208 pCRC | 12 (3-38) |  |  |  |  | 19 (5-57) |  |  |  |  |
|  |  |  | 6 (0-49) | 6 (0-43) | 15 (0-61) | 11 (1-61) |  | 9 (1-44) | 5 (2-69) | 22 (2-192) | 18 (2-185) |
|  | CD208 LM |  | 3 (0-128) | 4 (0-101) | 29 (3-146) | 6 (1-43) |  | 2 (1-21) | 7 (0-102) | 35 (2-112) | 10 (2-38) |
| Age,  below median | CD1a NAM | 1 (0-4)* |  |  |  |  | 0 (0-10) |  |  |  |  |
|  | CD1a pCRC |  | 8 (0-48)* | 14 (0-121)** | 6 (0-96)* | 1 (0-9) |  | 8 (0-62) | 10 (0-82) | 4 (0-49) | 0 (0-52) |
|  | CD1a LM |  | 2 (0-24) | 5 (0-108) | 1 (0-21) | 0 (0-3) |  | 4 (0-73) | 7 (0-151) | 4 (0-30) | 0 (0-8) |
|  | CD208 NAM | 16 (2-114) |  |  |  |  | 13 (4-37) |  |  |  |  |
|  | CD208 pCRC |  | 11 (1-43) | 8 (1-25) | 30 (5-132)** | 14 (2-111) |  | 14 (0-58) | 8 (0-50) | 25 (3-215) | 11 (0-83) |
|  | CD208 LM |  | 3 (0-44) | 4 (1-44) | 20 (4-230) | 6 (2-38) |  | 5 (0-73) | 8 (1-58) | 48 (7-232) | 19 (1-54) |
| Left -sided | CD1a NAM | 0 (0-4) |  |  |  |  | 0 (0-10) |  |  |  |  |
|  | CD1a pCRC |  | 5 (0-52) | 9 (0-121) | 4 (0-167) | 1 (0-106) |  | 7 (0-62) | 9 (0-82) | 3 (0-47) | 0 (0-6) |
|  | CD1a LM |  | 2 (0-31) | 4 (0-108) | 1 (0-23) | 0 (0-9) |  | 3 (0-73) | 7 (0-151) | 3 (0-73) | 0 (0-23) |
|  | CD208 NAM | 13 (2-114) |  |  |  |  | 14 (4-57) |  |  |  |  |
|  | CD208 pCRC |  | 6 (0-49) | 5 (0-43) | 18 (0-68) | 11 (1-58) |  | 11 (0-58) | 7 (0-69) | 22 (2-215) | 12 (0-83) |
|  | CD208 LM |  | 3 (0-128) | 4 (0-102) | 26 (3-230) | 6 (1-43) |  | 3 (0-73) | 7 (0-102) | 55 (2-232) | 15 (1-45) |
| Right -sided | CD1a NAM | 0 (0-2) |  |  |  |  | 0 (0-14) |  |  |  |  |
|  | CD1a pCRC |  | 9 (0-48) | 15 (0-55) | 5 (0-34) | 1 (0-5) |  | 7 (0-64) | 11 (2-130) | 3 (0-49) | 1 (0-52) |
|  | CD1a LM |  | 4 (0-19) | 7 (0-51) | 2 (0-20) | 0 (0-3) |  | 4 (0-7) | 7 (0-86) | 3 (0-13) | 0 (0-7) |
|  | CD208 NAM | 15 (3-38) |  |  |  |  | 18 (14-35) |  |  |  |  |
|  | CD208 pCRC |  | 12 (1-41) | 7 (2-17) | 30 (10-132) | 17 (2-111) |  | 9 (0-42) | 10 (2-64) | 32 (20-192) | 21 (3-185)* |
|  | CD208 LM |  | 2 (0-20) | 4 (1-40) | 26 (10-92) | 6 (3-21) |  | 4 (1-51) | 8 (4-49) | 38 (5-114) | 11 (2-54) |
| pCRC size,  above median | CD1a NAM | 0 (0-2) |  |  |  |  | 0 (0-14) |  |  |  |  |
|  | CD1a pCRC |  | 7 (0-52) | 13 (0-121) | 5 (0-167) | 0 (0-106) |  | 8 (0-64) | 10 (0-130) | 3 (0-49) | 0 (0-52) |
|  | CD1a LM |  | 3 (0-24) | 7 (0-108) | 2 (0-21) | 0 (0-3) |  | 3 (0-73) | 8 (0-120) | 5 (0-73) | 0 (0-23) |
|  | CD208 NAM | 12 (3-114) |  |  |  |  | 18 (5-38) |  |  |  |  |
|  | CD208 pCRC |  | 9 (0-41) | 7 (0-25) | 24 (5-95) | 9 (1-52) |  | 9 (0-49) | 7 (2-64) | 23 (3-192) | 15 (3-185) |
|  | CD208 LM |  | 4 (0-128) | 4 (0-44) | 28 (4-230) | 6 (1-34) |  | 4 (0-73) | 7 (0-102) | 44 (2-232) | 14 (2-54) |
| pCRC size,  below  median | CD1a NAM | 0 (0-4) |  |  |  |  | 0 (0-8) |  |  |  |  |
|  | CD1a pCRC |  | 5 (0-24) | 6 (0-46) | 3 (0-46) | 1 (0-9) |  | 6 (0-36) | 6 (0-61) | 3 (0-22) | 0 (0-2) |
|  | CD1a LM |  | 1 (0-31) | 3 (0-40) | 1 (0-23) | 0 (0-9) |  | 3 (0-60) | 3 (0-151) | 2 (0-30) | 0 (0-7) |
|  | CD208 NAM | 14 (2-49) |  |  |  |  | 13 (4-57) |  |  |  |  |
|  | CD208 pCRC |  | 6 (1-49) | 6 (0-43) | 16 (0-132) | 14 (3-111) |  | 16 (1-58)* | 8 (0-69) | 26 (2-215) | 12 (0-83) |
|  | CD208 LM |  | 3 (0-58) | 4 (0-102) | 26 (3-155) | 7 (1-43) |  | 2 (0-51) | 7 (1-49) | 37 (7-131) | 13 (1-40) |
| pCRC,  Grade 1 | CD1a NAM | 0 (0-0) |  |  |  |  | 0 (0-1) |  |  |  |  |
|  | CD1a pCRC |  | 7 (1-52) | 13 (1-105) | 5 (0-167) | 1 (0-106) |  | 5 (0-23) | 11 (0-37) | 5 (0-21) | 0 (0-2) |
|  | Cd1a LM |  | 2 (0-14) | 2 (1-31) | 1 (0-16) | 0 (0-2) |  | 3 (0-61) | 4 (0-151) | 4 (0-30) | 0 (0-7) |
|  | CD208 NAM | 14 (3-38) |  |  |  |  | 18 (4-38) |  |  |  |  |
|  | CD208 pCRC |  | 8 (1-41) | 7 (2-17) | 14 (6-95) | 13 (2-31) |  | 17 (4-58) | 7 (0-32) | 26 (4-58) | 12 (2-34) |
|  | CD208 LM |  | 3 (0-58) | 6 (0-102) | 30 (3-79) | 6 (5-40) |  | 4 (1-51) | 7 (0-49) | 36 (2-114) | 12 (2-40) |
| pCRC,  Grade 2 | CD1a NAM | 0 (0-4) |  |  |  |  | 0 (0-14) |  |  |  |  |
|  | CD1a pCRC |  | 5 (0-48) | 9 (0-121) | 4 (0-96) | 1 (0-9) |  | 8 (0-64) | 10 (0-130) | 3 (0-49) | 0 (0-52) |
|  | Cd1a LM |  | 2 (0-31) | 8 (0-108) | 2 (0-23) | 0 (0-9) |  | 3 (0-73) | 7 (0-97) | 3 (0-73) | 0 (0-23) |
|  | CD208 NAM | 13 (2-114) |  |  |  |  | 16 (5-57) |  |  |  |  |
|  | CD208 pCRC |  | 7 (0-49) | 5 (0-43) | 24 (0-132) | 13 (1-111) |  | 10 (0-57) | 7 (2-69) | 23 (3-215) | 18 (0-185) |
|  | CD208 LM |  | 4 (0-128) | 5 (0-44) | 21 (4-230) | 6 (1-43) |  | 2 (0-73) | 7 (1-102) | 44 (3-232) | 14 (1-54) |
| pCRC,  Grade 3 | CD1a NAM | 0 (0-1) |  |  |  |  | 0 (0-0) |  |  |  |  |
|  | CD1a pCRC |  | 7 (2-24) | 9 (4-22) | 3 (1-11) | 0 (0-2) |  | 3 (0-9) | 3 (0-11) | 1 (0-5) | 0 (0-0) |
|  | Cd1a LM |  | 3 (0-7) | 5 (0-21) | 1 (0-9) | 0 (0-3) |  | 3 (1-14) | 4 (1-67) | 2 (0-10) | 1 (0-2) |
|  | CD208 NAM | 31 (10-39) |  |  |  |  | 10 (5-13) |  |  |  |  |
|  | CD208 pCRC |  | 7 (2-33) | 13 (1-16) | 18 (7-43) | 11 (2-19) |  | 8 (1-37) | 7 (4-9) | 10 (2-38) | 4 (2-9) |
|  | CD208 LM |  | 3 (2-12) | 3 91-5) | 26 (7-52) | 10 (1-38) |  | 9 (2-20) | 13 (3-43) | 63 (23-131) | 15 (6-30) |
| N stage 0 | CD1a NAM | 0 (0-2) |  |  |  |  | 0 (0-0) |  |  |  |  |
|  | CD1a pCRC |  | 5 (0-48) | 13 (0-55) | 5 (0-46) | 1 (0-3) |  | 4 (0-23) | 7 (0-37) | 5 (0-21) | 0 (0-2) |
|  | CD1a LM |  | 2 (0-14) | 4 (0-32) | 1 (0-6) | 0 (0-2) |  | 2 (0-60) | 6 (0-151) | 5 (0-30) | 0 (0-7) |
|  | CD208 NAM | 17 (2-49) |  |  |  |  | 14 (4-38) |  |  |  |  |
|  | CD208 pCRC |  | 8 (1-39) | 5 (2-15) | 14 (6-39) | 9 (2-58) |  | 9 (0-58) | 6 (2-32) | 26 (2-58) | 12 (2-34) |
|  | CD208 LM |  | 4 (2-45) | 4 (2-23) | 19 (4-92) | 6 (2-40) |  | 3 (0-11) | 7 (1-17) | 29 (5-96) | 11 (4-38) |
| N stage 1 | CD1a NAM | 1 (0-4) |  |  |  |  | 0 (0-10) |  |  |  |  |
|  | CD1a pCRC |  | 6 (0-53) | 12 (0-121) | 6 (0-167) | 1 (0-106) |  | 7 (0-30) | 5 (0-79) | 2 (0-47) | 0 (0-2) |
|  | CD1a LM |  | 4 (0-17) | 9 (0-108) | 1 (0-21) | 0 (0-3) |  | 3 (0-73) | 6 (0-97) | 3 (0-21) | 0 (0-8) |
|  | CD208 NAM | 12 (3-114) |  |  |  |  | 16 (7-57) |  |  |  |  |
|  | CD208 pCRC |  | 7 (1-43) | 7 (0-25) | 25 (0-95) | 9 (2-61) |  | 11 (0-49) | 5 (0-15) | 21 (4-63) | 11 (2-28) |
|  | CD208 LM |  | 3 (0-128) | 5 (1-102) | 25 (6-155) | 6 (1-38) |  | 3 (1-73) | 7 (0-58) | 41 (2-232) | 19 (2-40) |
| N stage 2 | CD1a NAM | 0 (0-3) |  |  |  |  | 0 (0-14) |  |  |  |  |
|  | CD1a pCRC |  | 7 (0-24) | 8 (0-25) | 3 (0-35) | 0 (0-5) |  | 13 (1-64) | 19 (1-130) | 6 (0-49) | 1 (0-50) |
|  | CD1a LM |  | 2 (0-31) | 3 (0-59) | 1 (0-23) | 0 (0-9) |  | 4 (0-21) | 8 (0-67) | 3 (0-73) | 0 (0-23) |
|  | CD208 NAM | 16 (3-38) |  |  |  |  | 15 (5-44) |  |  |  |  |
|  | CD208 pCRC |  | 9 (0-49) | 6 (0-43) | 21 (5-132) | 14 (1-11) |  | 15 (3-57) | 10 (4-69)* | 32 (5-215) | 19 (0-185) |
|  | CD208 LM |  | 4 (0-31) | 4 (0-44) | 30 (3-230) | 7 (1-43) |  | 5 (0-51) | 7 (1-102) | 47 (3-131) | 12 (1-54) |
| Number of LM 1 | CD1a NM | 0 (0-4) |  |  |  |  | 0 (0-14) |  |  |  |  |
|  | CD1a pCRC |  | 7 (0-31) | 6 (0-121) | 5 (0-96) | 1 (0-9) |  | 7 (0-64) | 8 (0-130) | 2 (0-47) | 0 (0-6) |
|  | CD1a LM |  | 2 (0-24) | 2 (0-108) | 1 (0-21) | 0 (0-2) |  | 4 (0-73) | 8 (0-151) | 6 (0-73) | 0 (0-23) |
|  | CD208 NAM | 15 (6-114) |  |  |  |  | 16 (5-57) |  |  |  |  |
|  | CD208 pCRC |  | 9 (0-49) | 5 (0-25) | 24 (0-132) | 13 (1-111) |  | 13 (0-57) | 8 (0-69) | 27 (2-115) | 16 (2-185) |
|  | CD208 LM |  | 3 (0-45) | 4 (0-44) | 22 (3-230) | 6 (1-40) |  | 4 (0-73) | 7 (0-58) | 51 (2-232) | 18 (1-45) |
| Number of LM >1 | CD1a NAM | 0 (0-2) |  |  |  |  | 0 (0-10) |  |  |  |  |
|  | CD1a pCRC |  | 5 (0-52) | 12 (0-105) | 5 (0-167) | 1 (0-106) |  | 7 (0-59) | 10 (1-56) | 5 (0-49) | 0 (0-52) |
|  | CD1a LM |  | 2 (0-31) | 7 (0-40)* | 2 (0-23) | 0 (0-9) |  | 2 (0-39) | 3 (0-97) | 2 (0-22) | 0 (0-8) |
|  | CD208 NAM | 13 (2-49) |  |  |  |  | 16 (4-38) |  |  |  |  |
|  | CD208 pCRC |  | 8 (1-33) | 7 (1-43) | 19 (7-62) | 14 (2-61) |  | 10 (0-58) | 6 (2-32) | 23 (4-55) | 9 (0-51) |
|  | CD208 LM |  | 3 (0-128) | 4 (0-102) | 28 (6-146) | 6 (1-43) |  | 3 (1-21) | 7 (1-102) | 38 (3-131) | 11 (2-54) |
| Size of LM, below median | CD1a NAM | 0 (0-3) |  |  |  |  | 0 (0-14) |  |  |  |  |
|  | CD1a pCRC |  | 5 (0-48) | 8 (0-55) | 5 (0-46) | 1 (0-8) |  | 5 (0-64) | 9 (0-130) | 3 (0-25) | 0 (0-6) |
|  | CD1a LM |  | 2 (0-31) | 5 (0-59) | 1 (0-23) | 0 (0-9) |  | 4 (0-60) | 7 (0-151) | 3 (0-73) | 0 (0-23) |
|  | CD208 NAM | 12 (2-49) |  |  |  |  | 18 (4-56) |  |  |  |  |
|  | CD208 pCRC |  | 7 (1-49) | 7 (0-43) | 27 (0-132) | 15 (3-111)* |  | 12 (0-57) | 8 (2-64) | 25 (4-215) | 13 (0-187) |
|  | CD208 LM |  | 3 (0-128) | 5 (0-102) | 21 (3-230) | 5 (1-43) |  | 5 (0-51) | 9 (1-49)* | 62 (5-131)* | 13 (2-45) |
| Size of LM, above median | CD1a NAM | 0 (0-4) |  |  |  |  | 0 (0-10) |  |  |  |  |
|  | CD1a pCRC |  | 7 (0-52) | 12 (0-121) | 4 (0-167) | 0 (0-106) |  | 8 (0-59) | 8 (0-79) | 5 (0-49) | 0 (0-52) |
|  | Cd1a LM |  | 3 (0-17) | 6 (1-108) | 2 (0-21) | 0 (0-3) |  | 3 (0-73) | 7 (0-120) | 3 (0-22) | 0 (0-8) |
|  | CD208 NAM | 19 (3-114) |  |  |  |  | 14 (5-57) |  |  |  |  |
|  | CD208 pCRC |  | 8 (0-41) | 5 (0-25) | 19 (5-95) | 9 (1-31) |  | 9 (0-58) | 6 (0-69) | 22 (2-173) | 13 (2-51) |
|  | CD208 LM |  | 3 (0-45) | 4 (0-38) | 27 (4-155) | 9 (1-40)** |  | 2 (0-73) | 6 (0-102) | 24 (2-232) | 13 (1-54) |
| Grade LM1 | CD1a NAM | 0 (0-2) |  |  |  |  | 0 (0-10) |  |  |  |  |
|  | CD1a pCRC |  | 5 (0-52) | 11 (0-121) | 5 (0-167) | 0 (0-106) |  | 8 (0-62) | 11 (0-37) | 5 (0-21) | 0 (0-6) |
|  | CD1a LM |  | 2 (0-17) | 8 (0-108) | 2 (0-23)** | 1 (0-3) |  | 2 (0-60) | 7 (0-151) | 3 (0-30) | 0 (0-8) |
|  | CD208 NAM | 13 (3-144) |  |  |  |  | 17 (4-56) |  |  |  |  |
|  | CD208 pCRC |  | 7 (1-31) | 7 (1-25) | 19 (5-43) | 11 (2-61) |  | 9 (0-36) | 4 (0-32) | 22 (3-58) | 12 (2-34) |
|  | CD208 LM |  | 3 (0-58) | 5 (0-102) | 30 (10-146) | 7 (1-38) |  | 3 (0-51) | 5 (0-102) | 29 (2-114) | 11 (1-30) |
| Grade LM2 | CD1a NAM | 0 (0-4) |  |  |  |  | 0 (0-14) |  |  |  |  |
|  | CD1a pCRC |  | 6 (0-31) | 9 (0-46) | 4 (0-46) | 1 (0-9) |  | 7 (0-64) | 6 (0-130) | 3 (0-49) | 0 (0-52) |
|  | CD1a LM |  | 2 (0-31) | 3 (0-59) | 1 (0-20) | 0 (0-9) |  | 4 (0-73) | 6 (0-97) | 4 (0-73) | 0 (0-23) |
|  | CD208 NAM | 14 (2-49) |  |  |  |  | 16 (5-57) |  |  |  |  |
|  | CD208 pCRC |  | 7 (0-49) | 5 (0-43) | 21 (0-132) | 12 (1-111) |  | 14 (1-58) | 8 (2-69) | 24 (2-192) | 13 (0-185) |
|  | CD208 LM |  | 3 (0-128) | 5 (0-102) | 30 (10-146) | 7 (1-38) |  | 3 (1-73) | 7 (1-58) | 50 (7-232) | 18 (2-54) |
| LM margin R0 | CD1a NAM | 0 (0-4) |  |  |  |  | 0 (0-14) |  |  |  |  |
|  | CD1a pCRC |  | 8 (0-48) | 12 (0-121) | 4 (0-96) | 1 (0-9) |  | 7 (0-64) | 10 (0-130) | 4 (0-49) | 0 (0-52) |
|  | CD1a LM |  | 2 (0-31) | 5 (0-108) | 2 (0-23) | 0 (0-9) |  | 4 (0-73) | 8 (0-151) | 6 (0-73) | 1 (0-23)* |
|  | CD208 NAM | 16 (3-114) |  |  |  |  | 16 (4-57) |  |  |  |  |
|  | CD208 pCRC |  | 8 (0-49) | 7 (0-43) | 21 (0-132) | 13 (1-111) |  | 12 (0-58) | 7 (0-64) | 22 (3-215) | 12 (2-185) |
|  | CD208 LM |  | 3 (0-45) | 4 (0-44) | 27 (3-230) | 7 (1-43) |  | 3 (0-73) | 7 (0-102) | 43 (2-232) | 20 (1-54)** |
| LM margin R1 | CD1a NAM | 0 (0-1) |  |  |  |  | 0 (0-8) |  |  |  |  |
|  | CD1a pCRC |  | 4 (0-52) | 6 (0-105) | 5 (0-167) | 0 (0-106) |  | 4 (0-36) | 6 (0-21) | 3 (0-14) | 0 (0-2) |
|  | CD1a LM |  | 2 (0-14) | 6 (0-31) | 1 (0-16) | 1 (0-1) |  | 1 (0-28) | 2 (0-120) | 1 (0-13) | 0 (0-2)ws |
|  | CD208 NAM | 10 (2-29) |  |  |  |  | 14 (5-44) |  |  |  |  |
|  | CD208 pCRC |  | 6 (1-39) | 4 (1-19) | 14 (7-62) | 9 (4-61) |  | 9 (0-44) | 8 (2-69) | 26 (2-173) | 18 (0-39) |
|  | CD208 LM |  | 4 (0-128) | 5 (1-102) | 19 (6-146) | 6 (1-34) |  | 2 (1-20) | 6 (3-43) | 39 (5-131) | 8 (2-19) |

Notes: & - median (min-max) cell densities, * - p0.05, ** - p<0.01

Median age: synchronous =62, metachronous =64; median size for primary tumor: synchronous – 4.3cm, metachronous=3.5 cm; median size of liver metastasis: synchronous =2.0 cm, metachronous =2.7 cm.

Abbreviations: pCRC: primary colorectal cancer; LM: liver metastasis; NAM: non-tumor adjacent mucosa; TC: tumor center; IM: inner margin; OM: outer margin; PT: peritumoral zone; CHT: chemotherapy

Significant associations (others, n.s.):

1. Younger vs older age (synchronous cohort): higher CD1a in pCRC TC/IM/OM and higher CD208 in pCRC OM (p<0.05).
2. Right-sided vs left-sided pCRC (metachronous cohort): higher CD208 in pCRC PT (p<0.05).
3. Smaller vs larger pCRC size: higher CD208 in pCRC TC (p<0.05).
4. N2 stage vs N0-1 (metachronous cohort): higher CD208 in pCRC IM (p<0.05).
5. Number of LM >1 vs 1 (synchronous cohort): higher CD1a in LM IM (p<0.05).
6. Smaller LM size in the synchronous cohort was associated with higher CD208 in pCRC PT (p<0.05), whereas larger LM size was associated with higher CD208 in LM PT (p=0.01); in the metachronous cohort, smaller LM size was associated with higher CD208 in LM IM and OM (p<0.05).
7. LM grade 1 vs grade 2 (synchronous cohort): higher CD1a in LM OM (p<0.01).
8. LM margin R0 vs R1 (metachronous cohort) higher CD208 in LM in PT (p<0.01).

**Table S9.** Association between CD1a^+^ and CD208^+^ dendritic cells and chemotherapy before and after liver resection in CRC patients with synchronous and metachronous metastases

| **Variables** | **Cell type**  **and location** | **Synchronous** | | | | | **Metachronous** | | | | |
| --- | --- | --- | --- | --- | --- | --- | --- | --- | --- | --- | --- |
|  |  |  | **TC** | **IM** | **OM** | **PT** |  | **TC** | **IM** | **OM** | **PT** |
| CHT before liver surgery | CD1a NAM | 0 (0-3) |  |  |  |  | 0 (0-0) |  |  |  |  |
|  | CD1a pCRC |  | 5 (0-52) | 9 (0-121) | 4 (0-167) | 0 (0-106) |  | 6 (1-59) | 15 (0-56) | 14 (0-49) | 1 (0-52) |
|  | CD1a LM |  | 2 (0-31) | 5 (0-108) | 1 (0-21) | 0 (0-08) |  | 2 (0-60) | 9 (0-151) | 3 (0-30) | 1 (0-3) |
|  | CD208 NAM | 14 (2-49) |  |  |  |  | 20 (16-38) |  |  |  |  |
|  | CD208 pCRC |  | 7 (0-49) | 7 (0-44) | 20 (0-132) | 11 (*4-111) |  | 8 (3-16) | 5 (0-8) | 17 (10-58) | 11 (2-51) |
|  | CD208 LM |  | 3 (0-128) | 5 (0-102) | 29 (3-230) | 6 (0-43) |  | 3 (2-21) | 17 (8-102)** | 22 (3-55) | 19 (3-54) |
| CHT  after liver surgery | CD1a NAM | 0 (0-4) |  |  |  |  | 0 (0-14) |  |  |  |  |
|  | CD1a pCRC |  | 4 (0-20) | 8 (0-26) | 1 (0-26) | 0 (0-9) |  | 7 (0-64) | 9 (0-130) | 3 (0-47) | 0 (0-6) |
|  | CD1a LM |  | 2 (0-14) | 5 (1-32) | 1 (0-23) | 0 (0-2) |  | 4 (0-39) | 7 (0-97) | 6 (0-73) | 0 (0-23) |
|  | CD208 NAM | 13 (3-27) |  |  |  |  | 14 (4-57) |  |  |  |  |
|  | CD208 pCRC |  | 8 (1-17) | 5 (2-16) | 24 (7-68) | 14 (2-29) |  | 13 (0-58) | 7 (2-64) | 22 (3-215) | 12 (2-185) |
|  | CD208 LM |  | 4 (1-44) | 5 (0-38) | 25 (4-155) | 9 (2-38) |  | 3 (0-11) | 6 (0-24) | 43 (2-131) | 20 (1-45) |

Notes: & - median (min-max) cell densities; * - p0.05, ** - p<0.01

Abbreviations: pCRC: primary colorectal cancer; LM: liver metastasis; NAM: non-tumor adjacent mucosa; TC: tumor center; IM: inner margin; OM: outer margin; PT: peritumoral zone; CHT: chemotherapy

Significant associations (others, n.s.):

1. CHT before vs after liver surgery (metachronous cohort): higher CD208 in LM IM (p<0.01)

**Table S10.** Association between CD1a^+^ and CD208^+^ dendritic cells and FOLFOX-based chemotherapy versus other in CRC patients with synchronous and metachronous metastases

| **Chemotherapy**  **regimen** | **Cell type**  **and location** | **Synchronous** | | | | | **Metachronous** | | | | |
| --- | --- | --- | --- | --- | --- | --- | --- | --- | --- | --- | --- |
|  |  |  | **TC** | **IM** | **OM** | **PT** |  | **TC** | **IM** | **OM** | **PT** |
| FOLFOX | CD1a NAM | 0 (0-1) |  |  |  |  | 0 (0-10) |  |  |  |  |
|  | CD1a pCRC |  | 5 (0-52) | 10 (0-121) | 4 (0-167) | 1 (0-106) |  | 7 (0-36) | 5 (0-82) | 3 (0-25) | 0 (0-4) |
|  | CD1a LM |  | 2 (0-31) | 5 (0-108) | 1 (0-21) | 0 (0-9) |  | 4 (0-22) | 6 (0-67) | 3 (0-73) | 0 (0-23) |
|  | CD208 NAM | 12 (2-49) |  |  |  |  | 14 (5-56) |  |  |  |  |
|  | CD208 pCRC |  | 5 (1041) | 7 (2043) | 24 (8-132)* | 15 (2-111)* |  | 10 (0-58) | 6 (2-69) | 21 (9-215) | 12 (2-83) |
|  | CD208 LM |  | 4 (0-128) | 5 (0-102) | 29 (3-230) | 5 (1-43) |  | 5 (0-21) | 8 (1-43) | 58 (7-131) | 9 (1-45) |
| Other | CD1a NAM | 0 (0-4) |  |  |  |  | 0 (0-14) |  |  |  |  |
|  | CD1a pCRC |  | 7 (0-31) | 6 (0-53) | 5 (0-34) | 0 (0-9) |  | 7 (0-64) | 11 (0-130) | 5 (0-49) | 0 (0-52) |
|  | CD1a LM |  | 2 (0-18) | 6 (0-51) | 1 (0-23) | 0 (0-2) |  | 2 (0-60) | 8 (0-151) | 4 (0-30) | 0 (0-7) |
|  | CD208 NAM | 14 (3-114) |  |  |  |  | 17 (4-57) |  |  |  |  |
|  | CD208 pCRC |  | 6 (0-49) | 4 (0-16) | 15 (0-39) | 8 (1-54) |  | 11 (0-42) | 7 (0-64) | 25 (2-191) | 13 (0-185) |
|  | Cd208 LM |  | 2 (0-45) | 4 (0-41) | 21 (6-155) | 7 (1-40) |  | 2 (0-51) | 6 (0-102) | 35 (2-114) | 14 (2-54) |

Notes: & - median (min-max) cell densities; * - p<0.05

Abbreviations: pCRC: primary colorectal cancer; LM: liver metastasis; NAM – non-tumor adjacent mucosa; TC: tumor center; IM: inner margin; OM: outer margin; PT: peritumoral zone; CHT: chemotherapy

Significant associations (others, n.s.):

1. FOLFOX regimen vs others (synchronous cohort): higher CD208 in LM OM and in LM PT (p<0.05).

**Table S11.** Spearman’s rank correlation between CD208^+^ DCs and T-cells

|  | Synchronous | | | | | | | | |
| --- | --- | --- | --- | --- | --- | --- | --- | --- | --- |
|  | **CD208**  **NAM** | **CD208**  **pCRC TC** | **CD208**  **pCRC IM** | **CD208**  **pCRC OM** | **CD208**  **pCRC PT** | **CD208**  **LM TC** | **CD208**  **LM IM** | **CD208**  **LM OM** | **CD208**  **LM PT** |
| **CD3**  **NAM** | 0.37* |  |  |  |  |  |  |  |  |
| **CD3**  **pCRC TC** |  | 0.58* |  |  |  |  |  |  |  |
| **CD3**  **pCRC IM** |  |  | 0.47* |  |  |  |  |  |  |
| **CD3**  **pCRC OM** |  |  |  | 0.48* |  |  |  |  |  |
| **CD3**  **pCRC PT** |  |  |  |  | 0.38* |  |  |  |  |
| **CD3**  **LM TC** |  |  |  |  |  | 0.67* |  |  |  |
| **CD3**  **LM IM** |  |  |  |  |  |  | 0.63* |  |  |
| **CD3**  **LM OM** |  |  |  |  |  |  |  | 0.65* |  |
| **CD3**  **LM PT** |  |  |  |  |  |  |  |  | 0.57* |
| **CD8**  **NAM** | 0.07 |  |  |  |  |  |  |  |  |
| **CD8**  **pCRC TC** |  | 0.47* |  |  |  |  |  |  |  |
| **CD8**  **pCRC IM** |  |  | 0.42* |  |  |  |  |  |  |
| **CD8**  **pCRC OM** |  |  |  | 0.20 |  |  |  |  |  |
| **CD8**  **pCRC PT** |  |  |  |  | 0.18 |  |  |  |  |
| **CD8**  **LM TC** |  |  |  |  |  | 0.64* |  |  |  |
| **CD8**  **LM IM** |  |  |  |  |  |  | 0.54* |  |  |
| **CD8**  **LM OM** |  |  |  |  |  |  |  | 0.49* |  |
| **CD8**  **LM PT** |  |  |  |  |  |  |  |  | 0.37* |
| **CD45RO**  **NAM** | 0.24 |  |  |  |  |  |  |  |  |
| **CD45**  **pCRC TC** |  | 0.52* |  |  |  |  |  |  |  |
| **CD45**  **pCRC IM** |  |  | 0.32* |  |  |  |  |  |  |
| **CD45**  **pCRC OM** |  |  |  | 0.58* |  |  |  |  |  |
| **CD45**  **pCRC PT** |  |  |  |  | 0.41* |  |  |  |  |
| **CD45**  **LM TC** |  |  |  |  |  | 0.60* |  |  |  |
| **CD45**  **LM IM** |  |  |  |  |  |  | 0.52* |  |  |
| **CD45**  **LM OM** |  |  |  |  |  |  |  | 0.46* |  |
| **CD45**  **LM PT** |  |  |  |  |  |  |  |  | 0.55* |
|  | Metachronous | | | | | | | | |
|  | **CD208**  **NAM** | **CD208**  **pCRC TC** | **CD208**  **pCRC IM** | **CD208**  **pCRC OM** | **CD208**  **pCRC PT** | **CD208**  **LM TC** | **CD208**  **LM IM** | **CD208**  **LM OM** | **CD208**  **LM PT** |
| **CD3**  **NAM** | 0.32* |  |  |  |  |  |  |  |  |
| **CD3**  **pCRC TC** |  | 0.44* |  |  |  |  |  |  |  |
| **CD3**  **pCRC IM** |  |  | 0.42* |  |  |  |  |  |  |
| **CD3**  **pCRC OM** |  |  |  | 0.52* |  |  |  |  |  |
| **CD3**  **pCRC PT** |  |  |  |  | 0.48* |  |  |  |  |
| **CD3**  **LM TC** |  |  |  |  |  | 0.50* |  |  |  |
| **CD3**  **LM IM** |  |  |  |  |  |  | 0.40* |  |  |
| **CD3**  **LM OM** |  |  |  |  |  |  |  | 0.62* |  |
| **CD3**  **LM PT** |  |  |  |  |  |  |  |  | 0.59* |
| **CD8**  **NAM** | 0.28 |  |  |  |  |  |  |  |  |
| **CD8**  **pCRC TC** |  | 0.31 |  |  |  |  |  |  |  |
| **CD8**  **pCRC IM** |  |  | 0.24 |  |  |  |  |  |  |
| **CD8**  **pCRC OM** |  |  |  | 0.23 |  |  |  |  |  |
| **CD8**  **pCRC PT** |  |  |  |  | 0.30* |  |  |  |  |
| **CD8**  **LM TC** |  |  |  |  |  | 0.50* |  |  |  |
| **CD8**  **LM IM** |  |  |  |  |  |  | 0.45* |  |  |
| **CD8**  **LM OM** |  |  |  |  |  |  |  | 0.44* |  |
| **CD8**  **LM PT** |  |  |  |  |  |  |  |  | 0.33* |
| **CD45RO**  **NAM** | 0.00 |  |  |  |  |  |  |  |  |
| **CD45**  **pCRC TC** |  | 0.32* |  |  |  |  |  |  |  |
| **CD45**  **pCRC IM** |  |  | 0.31* |  |  |  |  |  |  |
| **CD45**  **pCRC OM** |  |  |  | 0.39* |  |  |  |  |  |
| **CD45**  **pCRC PT** |  |  |  |  | 0.48* |  |  |  |  |
| **CD45**  **LM TC** |  |  |  |  |  | 0.35* |  |  |  |
| **CD45**  **LM IM** |  |  |  |  |  |  | 0.15 |  |  |
| **CD45**  **LM OM** |  |  |  |  |  |  |  | 0.54* |  |
| **CD45**  **LM PT** |  |  |  |  |  |  |  |  | 0.52* |

*** - p<0.05**

**Abbreviations:** pCRC: primary colorectal cancer; LM: liver metastasis; NAM – non-tumor adjacent mucosa; TC: tumor center; IM: inner margin; OM: outer margin; PT: peritumoral zone

**Table S12**. Spearman’s rank correlation between CD1a^+^ DCs and T-cells

|  | Synchronous | | | | | | | | |
| --- | --- | --- | --- | --- | --- | --- | --- | --- | --- |
|  | **CD1a**  **NAM** | **CD1a**  **pCRC TC** | **CD1a**  **pCRC IM** | **CD1a**  **pCRC OM** | **CD1a**  **pCRC PT** | **CD1a**  **LM TC** | **CD1a**  **LM IM** | **CD1a**  **LM OM** | **CD1a**  **LM PT** |
| **CD3**  **NAM** | 0.04 |  |  |  |  |  |  |  |  |
| **CD3**  **pCRC TC** |  | 0.30* |  |  |  |  |  |  |  |
| **CD3**  **pCRC IM** |  |  | 0.08 |  |  |  |  |  |  |
| **CD3**  **pCRC OM** |  |  |  | 0.15 |  |  |  |  |  |
| **CD3**  **pCRC PT** |  |  |  |  | 0.38* |  |  |  |  |
| **CD3**  **LM TC** |  |  |  |  |  | 0.53* |  |  |  |
| **CD3**  **LM IM** |  |  |  |  |  |  | 0.34* |  |  |
| **CD3**  **LM OM** |  |  |  |  |  |  |  | 0.41* |  |
| **CD3**  **LM PT** |  |  |  |  |  |  |  |  | 0.23 |
| **CD8**  **NAM** | -0.25 |  |  |  |  |  |  |  |  |
| **CD8**  **pCRC TC** |  | 0.24 |  |  |  |  |  |  |  |
| **CD8**  **pCRC IM** |  |  | 0.06 |  |  |  |  |  |  |
| **CD8**  **pCRC OM** |  |  |  | 0.17 |  |  |  |  |  |
| **CD8**  **pCRC PT** |  |  |  |  | 0.35* |  |  |  |  |
| **CD8**  **LM TC** |  |  |  |  |  | 0.55* |  |  |  |
| **CD8**  **LM IM** |  |  |  |  |  |  | 0.30* |  |  |
| **CD8**  **LM OM** |  |  |  |  |  |  |  | 0.18 |  |
| **CD8**  **LM PT** |  |  |  |  |  |  |  |  | 0.17 |
| **CD45RO**  **NAM** | -0.06 |  |  |  |  |  |  |  |  |
| **CD45**  **pCRC TC** |  | 0.04 |  |  |  |  |  |  |  |
| **CD45**  **pCRC IM** |  |  | 0.03 |  |  |  |  |  |  |
| **CD45**  **pCRC OM** |  |  |  | 0.07 |  |  |  |  |  |
| **CD45**  **pCRC PT** |  |  |  |  | 0.14 |  |  |  |  |
| **CD45**  **LM TC** |  |  |  |  |  | 0.43* |  |  |  |
| **CD45**  **LM IM** |  |  |  |  |  |  | 0.29* |  |  |
| **CD45**  **LM OM** |  |  |  |  |  |  |  | 0.28* |  |
| **CD45**  **LM PT** |  |  |  |  |  |  |  |  | 0.26 |
|  | Metachronous | | | | | | | | |
|  | **CD1a**  **NAM** | **CD1a**  **pCRC TC** | **CD1a**  **pCRC IM** | **CD1a**  **pCRC OM** | **CD1a**  **pCRC PT** | **CD1a**  **LM TC** | **CD1a**  **LM IM** | **CD1a**  **LM OM** | **CD1a**  **LM PT** |
| **CD3**  **NAM** | 0.19 |  |  |  |  |  |  |  |  |
| **CD3**  **pCRC TC** |  | 0.23 |  |  |  |  |  |  |  |
| **CD3**  **pCRC IM** |  |  | 0.13 |  |  |  |  |  |  |
| **CD3**  **pCRC OM** |  |  |  | 0.19 |  |  |  |  |  |
| **CD3**  **pCRC PT** |  |  |  |  | 0.26 |  |  |  |  |
| **CD3**  **LM TC** |  |  |  |  |  | 0.21 |  |  |  |
| **CD3**  **LM IM** |  |  |  |  |  |  | 0.31* |  |  |
| **CD3**  **LM OM** |  |  |  |  |  |  |  | 0.42* |  |
| **CD3**  **LM PT** |  |  |  |  |  |  |  |  | 0.44* |
| **CD8**  **NAM** | 0.20 |  |  |  |  |  |  |  |  |
| **CD8**  **pCRC TC** |  | 0.02 |  |  |  |  |  |  |  |
| **CD8**  **pCRC IM** |  |  | -0.17 |  |  |  |  |  |  |
| **CD8**  **pCRC OM** |  |  |  | -0.14 |  |  |  |  |  |
| **CD8**  **pCRC PT** |  |  |  |  | -0.05 |  |  |  |  |
| **CD8**  **LM TC** |  |  |  |  |  | 0.16 |  |  |  |
| **CD8**  **LM IM** |  |  |  |  |  |  | 0.15 |  |  |
| **CD8**  **LM OM** |  |  |  |  |  |  |  | 0.32* |  |
| **CD8**  **LM PT** |  |  |  |  |  |  |  |  | 0.30 |
| **CD45RO**  **NAM** | 0.06 |  |  |  |  |  |  |  |  |
| **CD45**  **pCRC TC** |  | 0.20 |  |  |  |  |  |  |  |
| **CD45**  **pCRC IM** |  |  | 0.18 |  |  |  |  |  |  |
| **CD45**  **pCRC OM** |  |  |  | 0.19 |  |  |  |  |  |
| **CD45**  **pCRC PT** |  |  |  |  | 0.31* |  |  |  |  |
| **CD45**  **LM TC** |  |  |  |  |  | 0.20 |  |  |  |
| **CD45**  **LM IM** |  |  |  |  |  |  | 0.50* |  |  |
| **CD45**  **LM OM** |  |  |  |  |  |  |  | 0.57* |  |
| **CD45**  **LM PT** |  |  |  |  |  |  |  |  | 0.42* |

*** - p<0.05**

**Abbreviations:** pCRC: primary colorectal cancer; LM: liver metastasis; NAM – non-tumor adjacent mucosa; TC: tumor center; IM: inner margin; OM: outer margin; PT: peritumoral zone
